# TGF‐β1 regulates the lncRNA transcriptome of ovarian granulosa cells in a transcription activity‐dependent manner

**DOI:** 10.1111/cpr.13336

**Published:** 2022-09-20

**Authors:** Qiqi Li, Yangan Huo, Siqi Wang, Liu Yang, Qifa Li, Xing Du

**Affiliations:** ^1^ Laboratory of Statistical Genetics and Epigenetics, College of Animal Science and Technology Nanjing Agricultural University Nanjing China

## Abstract

**Objectives:**

Transforming growth factor β1 (TGF‐β1), an essential cytokine belongs to TGF‐β superfamily, is crucial for female fertility. Increasing evidence show that long noncoding RNAs (lncRNAs) influence the state of granulosa cells (GCs). This study aimed to detect the effects of TGF‐β1 on the lncRNA transcriptome, and investigate whether lncRNAs mediate the functions of TGF‐β1 in GCs.

**Material and Methods:**

RNA‐seq and bioinformatics analyses were performed to identify and characterize the differentially expressed lncRNAs (DElncRNAs). The regulatory mechanism of TGF‐β1 to lncRNA transcriptome was analyzed by chromatin immunoprecipitation. The effects of lncRNAs on the antiapoptotic and proproliferative functions of TGF‐β1 were examined by morphological analysis, fluorescence‐activated cell sorting, Cell Counting Kit‐8, and Western blot.

**Results:**

A total of 72 DElncRNAs highly sensitive to TGF‐β1 were identified with the criteria of |log_2_(fold chage)| ≥ 3 and false discovery rate < 0.05. Functional assessment showed that DElncRNAs were enriched in TGF‐β, nuclear factor kappa B, p53, and Hippo pathways which are crucial for the normal state and function of GCs. Importantly, SMAD4 is essential for the regulation of TGF‐β1 to lncRNA transcriptome. In vitro studies confirmed that TGF‐β1 induced *TEX14‐IT1* transcription in a SMAD4‐dependent manner, and TEX14‐IT1 mediated the antiapoptotic and proproliferative effects of TGF‐β1 in GCs.

**Conclusions:**

Our findings demonstrate that TGF‐β1 alters lncRNA transcriptome in a SMAD4‐dependent manner, and highlight that lncRNAs mediate the functions of TGF‐β1 in GCs, which contribute to a better understanding of the epigenetic regulation of female fertility.

## Graphical abstract

The crosstalks between transforming growth factor β1 (TGF‐β1) and noncoding RNAs influence the normal states and functions of ovarian granulosa cells (GCs). RNA‐seq and bioinformatics analysis showed that TGF‐β1 regulates the long noncoding RNA (lncRNA) transcriptome of porcine GCs in a SMAD4‐dependent manner, which is also universal for the TGF‐β1‐induced transcriptomic alteration. In vitro studies confirmed that lncRNAs, such as TEX14‐IT1, mediated the antiapoptotic and proproliferative functions of TGF‐β1 in GCs. Our current study provides a panoramic view of lncRNA transcriptome and highlight the potential role of lncRNAs in mediating the biological functions of canonical TGF‐β signaling pathway, which contributes to a better understanding of the epigenetic regulation of female fertility.

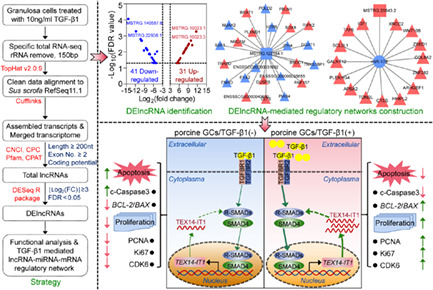

## 1 INTRODUCTION

Normal follicular development and ovulation are essential for the maintenance of the reproductive processes in female mammals.^1^ However, only a few follicles can mature and eventually ovulate, while more than 99% of follicles undergo atresia and degradation at each stage during follicular development, which is also known as the dominant follicle selection.^2^, ^3^ This process causes a great waste of biological resources from the perspective of reproductive biology, and severe follicular atresia leads to preantral follcile degradation, premature follicle failure, polycystic ovary syndrome, and even infertility.^4^, ^5^, ^6^ Although the initiation mechanism and regulatory modes of follicular atresia remain largely unknown, it is generally believed mainly caused by granulosa cell (GC) apoptosis and nonapoptotic programmed cell death,^7^, ^8^ which are controlled by a complicated regulatory network composed of multiple crucial factors and signaling pathways.^9^, ^10^ Among which, transforming growth factor β1 (TGF‐β1) and TGF‐β signaling pathways are the pivotal regulators of the GC state and follicle fate.^11^, ^12^

TGF‐β1, a crucial multifunctional cytokine and essential ligand of the TGF‐β superfamily signaling pathways, is involved in the regulation of multiple crucial biological events and plays important roles in various physiological and pathological processes.^13^, ^14^ Regardless of its complicated functions, TGF‐β1‐mediated signal transduction into the nucleus is quite simple, which can be divided into two different pathways: canonical (SMADs‐dependent) and noncanonical (SMADs‐independent) pathways.^15^, ^16^ In the canonical TGF‐β signaling pathway, the extracellular ligands (e.g., TGF‐β1) with signals specifically bind to and activate type II and type I receptors (e.g., TGFBR2 and TGFBR1) in the cell membrane, which further phosphorylate activates R‐SMADs (e.g., SMAD2/3) and shuttles into the nucleus by forming a heteromeric complex with SMAD4 (the only co‐SMAD). In the nucleus, the R‐SMADs/SMAD4 transcription complex with signals regulates the transcription of downstream target genes (coding and noncoding) with or without the help of coregulators depending on the cell type and biological contexts.^17^, ^18^, ^19^

TGF‐β1 is highly expressed in the reproductive system, and the activation of the TGF‐β signaling pathways is necessary for the female fertility, mainly by inhibiting follicular atresia and maintaining the normal states and functions of GCs.^20^, ^21^ For example, in vivo experiments using the *Tgf‐β1* knockout female mice model revealed that the genetic deficiency of *Tgf‐β1* leads to an abnormal estrous cycle, incomplete oocyte development, early embryo arrest, and severe infertility.^21^ In addition, multiple studies on GCs cultured in vitro have demonstrated that TGF‐β1 addition induces the proliferation and viability, while inhibits the apoptosis of GCs.^22^ While, the opposite phenomena were occurred in GCs when TGF‐β1 knockdown or TGF‐β signaling pathway inhibitor (SB431542, LY2157299) treatment.^23^, ^24^ Recent studies with the in‐depth investigation on noncoding RNAs have identified a variety of noncoding RNAs (miRNAs and circRNAs) that mediate the regulation of TGF‐β1 during follicular development and atresia.^25^, ^26^

In our previous study, 19 differentially expressed miRNAs (DEmiRNAs) involved in the regulation of GC state (apoptosis, proliferation, and viability) and function (E2 secretion) were identified after treatment with 10 ng/ml of TGF‐β1.^11^ However, it remains unclear whether long noncoding RNAs (lncRNAs), another important type of noncoding RNA, mediate the functions of TGF‐β1 in ovarian GCs. The aim of this study is to accurately identify the DElncRNAs highly sensitive to TGF‐β1 in GCs, establish the TGF‐β1‐mediated lncRNA regulatory networks, and reveal the lncRNA‐dependent regulatory mechanism of TGF‐β1 during follicular development and female fertility.

## 2 MATERIALS AND METHODS

### 2.1 Animals and ethics statement

A total of 105 healthy, sexually mature, and non‐estrus Duroc–Landrace–Yorkshire sows (*n* = 105; average 180 days and average mess 110 kg) were randomly selected from Zhushun Biological Technology Co. for bilateral ovaries collection and GCs isolation. The studied sows were healthy, unstimulated, taken care, and slaughtered according to the guideline of animal welfare regulations. All the animal‐involved experiments in this study were reviewed, approved, and supervised by the Animal Ethics Committee of Nanjing Agricultural University, China (NJAU. No20223024059) and performed according to the Regulations for the Administration of Affairs Concerning Experimental Animals (No. 2 of the State Science and Technology Commission, 11/14/1988, China).

### 2.2 Cell culture and treatment

Porcine GCs from fresh nonatretic follicles (3–5 mm diameter) were isolated and cultured in vitro as described previously.^3^ Briefly, porcine GCs were extracted by using a syringe with 22‐gauge needle and seeded into 6‐ and 12‐well plates filled with DEME‐F12 medium (#11320033; Invitrogen) containing 10% fetal bovine serum (FBS; #30044333; Gibco), and 1% streptomycin–penicillin, which were then placed in a 37°C incubator with humid atmosphere and 5% CO_2_. After incubation for 48 h, cells were firmly attached and prepared for the further in vitro treatment. All the cells used in this study were tested to be uncontaminated and mycoplasma‐negative. For TGF‐β1 and SB431542 treatment, the culture medium were replaced with fresh FBS‐free medium for 10 h, and then TGF‐β1 (#P01137; Novoprotein) and SB431542 (#S1067; Selleck) were added into the culture medium to the final concentration of 10 ng/ml and 10 μM, respectively. For cell transfection, 50 nM small interfering RNAs (siRNAs) and 2.5 μg plasmids were transfected into GCs using Lipofectamine 3000 reagent (#L13778‐150; Invitrogen). The oligonucleotides used in this study were designed and synthesized by GenePharma, which were listed in Table S1.

### 2.3 RNA extraction, cDNA libraries preparation, and lncRNA sequencing

After treatment for 24 h, the total RNA from porcine GCs under different treatment were isolated and purified by using TRIzol reagent (#R401‐01; Vazyme Biotech Co., Ltd.). RNA degradation and contamination was monitored on 1.5% agarose gels. The quality and quantity of the purified RNA were analyzed by NanoDrop 3000 system (Thermo Fisher Scientific), and the integrity and purity of each RNA sample was detected by an Agilent 2100 Bioanalyzer (Agilent Technologies). The cDNA libraries preparation were performed as described previously^27^ and sent to BioMarker Technologies Co., Ltd for lncRNA sequencing. After removing ribosomal RNA (rRNA) by Ribo‐Zero rRNA Removal Kit (Epicentre), the purified RNA was reverse‐transcribed into cDNA and digested into 150–200 bp fragments for cDNA libraries construction. Then, Illumina HiSeq3000 platform was applied for sequencing and paired‐end reads generation. High‐quality clean reads after removing adapter sequences and low‐quality reads (*Q* ≤ 20% and > 50%) were obtained for further analyses. The total clean reads were mapped with the background of *Sus Scrofa* RefSeq (*Sscrofa* 11.1) using TopHat v2.0.9,^28^ and the transcripts were assembled and merged through Cufflinks^29^ with reference annotation based transcript method.

### 2.4 Identification and characterization of DElncRNAs

The lncRNA profiles were identified by screening the merged transcript sets with the following five criteria: (1) transcript length ≥ 200 nt; (2) transcripts with exon numbers ≥2; (3) filtering out the transcripts that overlapped with exon regions annotated as coding genes in the database; (4) transcripts with an FPKM (fragments per kilobase of exons per million mapped fragments) ≥ 0.1 in at least one group; (5) transcripts with low protein‐coding potential were identified by four different algorithms (CNCI v2.0,^30^ CPAT v2.0,^31^ CPC‐0.9‐r2,^32^ and Pfam v1.3^33^). After stringent and systematic filtering, the remaining transcripts were considered as putative lncRNAs. To detect the character differences between lncRNAs and mRNAs, their expression levels, transcript length, exon numbers, open reading frame (ORF) length, and chromosome distribution were analyzed and compared. After quantile normalization, the FPKM value of each lncRNA was calculated and their changes were transformed with log2. Besides, the significance of each lncRNA between different groups was computed and adjusted as false discovery rate (FDR). The DElncRNAs highly sensitive to TGF‐β1 were identified using the DESeq R package v1.10.1 with the cut‐off criteria: |log_2_(fold change)| ≥ 3 (fold change ≤ 0.125 or ≥8) and adjusted FDR value ≤ 0.05.

### 2.5 Functional analyses of DElncRNAs

In order to investigate the potential function of DElncRNAs, we have predicted their *trans*‐ and *cis*‐target genes based on different principles, and further intersected with DEmRNA profile to improve the accuracy of prediction. The *trans*‐target genes were identified based on the following cut‐off criteria: (1) complementary sequences between DElncRNAs and mRNAs with normalized free energy < −0.1 by using LncTar software; (2) the Pearson correlation coefficient between DElncRNAs and mRNAs, |*r*| > 0.95. In addition, the *cis*‐target genes were classified as the DEmRNAs transcribed from the region located ~100 kb upstream and downstream of the DElncRNAs by Perl. Finally, the targets of DElncRNAs were considered as the integrity of *trans*‐ and *cis*‐target genes. In addition, Data for Annotation, Visualization and Integrated Discovery (DAVID v6.8)^34^ was performed for Gene Ontology (GO) and Kyoto Encyclopedia of Genes and Genomes (KEGG) pathway enrichment analyses to analyse the potential role and function of the identified DElncRNAs based on their potential targets. Only the GO terms and KEGG terms with adjusted *p* < 0.05 were considered as significantly enriched.

### 2.6 Regulatory networks construction and functional analysis

For the effective DElncRNAs‐mediated protein–protein interaction (PPI) network establishment, all the function‐known protein‐coding targets of DElncRNAs were selected and the PPI network was constructed as described previously.^35^ In brief, STRING v11.0 database^36^ was used to generate the interactions with the following basic settings: (1) the minimum required interaction score ≥ 0.9 [0–1]; (2) the interaction protein amount ≥ 1. The PPI network was visualized by using Cytoscape v3.7.2 software, and the nodes with higher degrees (top 5%) were considered as hub genes through Cytohubba function. The characters of the PPI network were obtained from STRING with “Analysis” function based on the interactions within the network, including the enrichment *p* value, the average node degree (AND), and the average local clustering coefficient (ALCC). The different modules in PPI network were identified by MCODE function. The potential functions of different modules were analyzed using DAVID v6.8. For DElncRNA–DEmRNA pathway function regulatory network construction, all the targets (both *trans*‐ and *cis*‐targets) of DElncRNAs were intersected with the DEmRNAs to obtain the interactions, and the significant enriched signaling pathways and potential functions were analyzed by using KEGG and GO annotation. To establish the TGF‐β1‐mediated DElncRNA–DEmiRNA–DEmRNA network, DElncRNAs and DEmiRNAs from the RNA‐seq data were selected to predict the DElncRNA–DEmiRNA negative regulatory interactions using RNAhybrid (https://bibiserv.cebitec.uni-bielefeld.de/rnahybrid/) and miRDB (http://mirdb.org/). Finally, the network was established with the DEmiRNA–DEmRNA complementary pairs.

### 2.7 Rapid amplification of cDNA end

The full‐length sequence of pig TEX14‐IT1 was identified by using the rapid amplification of cDNA end (RACE) with SMARTer RACE 5′/3′ Kit (#634858; Clontech Laboratories, Inc.) according to the manufacturer's instruction. Briefly, 4 μg high quality total RNA from porcine GCs was used for RACE‐Ready cDNA synthesis, and the 5′‐ and 3′‐end of TEX14‐IT1 were amplified with gene specific primers. The gene specific primers designed for RACE were listed below: 5′‐GSP, 5′‐CGGGTGCTCTACACGTTCAGAGAAACT‐3′; 3′‐GSP, 5′‐AGCCCGGCTCTTGGTGTTGCCTGTGC‐3′. The corresponding products were analyzed by electrophoresis on a 1.5% agarose gel, and the clear DNA bands were collected, purified, and cloned into pClone007 vector. The terminals of TEX14‐IT1 were verified by Sanger sequencing and mapped to the *Sus scrofa* RefSeq 11.1.

### 2.8 Quantitative real‐time PCR

To confirm the accuracy of RNA‐seq and detect the expression pattern of crucial genes, quantitative real‐time polymerase chain reaction (qRT‐PCR) was performed as previously described.^37^ Briefly, 1 μg total RNA extracted from GCs was reverse‐transcribed into cDNA using HiScript III RT SuperMix for qPCR with gDNA wiper (#R323‐01; Vazyme Biotech Co., Ltd.). qRT‐PCR assays were performed using the SYBR Green Master Mix (#Q111‐02; Vazyme Biotech Co., Ltd.) in a StepOne Plus Q7 detection system (Applied Biosystems) with three independent biological replicates. The original data were analyzed with 2^−∆∆*C*^_T_ method and the relative expression levels of lncRNAs and protein‐coding genes were normalized to *GAPDH*. For miRNA expression levels detection, stem‐loop primers were designed to synthesize cDNA, and specific primers targeting mature miRNAs were designed for qRT‐PCR. The expression levels of mature miRNAs were normalized to *U6* small nuclear RNA (*U6*). qRT‐PCR assays were performed in triplicate. The primers used here were listed in Table S2.

### 2.9 Bioinformatics analysis

In this study, three online tools were used to predict the candidate core promoters of DElncRNAs, including Promoter 2.0 (http://www.cbs.dtu.dk/services/Promoter/), BDGP (https://www.fruitfly.org/seq_tools/promoter.html), and Softberry (http://www.softberry.com/). For transcription factor binding motifs analyses, two database, including JASPAR (http://jaspar.genereg.net/) and FIMO (http://meme-suite.org/tools/fimo) were utilized. The genomic location, H3K27Ac modification, and mammalian conservation of DElncRNAs were referred to three well‐known database, which are NCBI (https://www.ncbi.nlm.nih.gov/), Ensembl (ensembl.org/index.html), and UCSC (http://genome.ucsc.edu/). The subcellular location of DElncRNAs was predicted by Lnclocator, an online prediction system (http://www.csbio.sjtu.edu.cn/bioinf/lncLocator/).

### 2.10 Plasmids construction and luciferase activity assay

The overexpression plasmid pcDNA3.1‐SMAD4 was kindly provided by Jiying Liu from Jiangsu University of Science and Technology. The promoter fragments of pig *TEX14* and *TEX14‐IT1* with the wild‐type SMAD4 binding elements (SBEs) were synthesized at Sangon Biotechnologies and cloned into the pGL3‐Basic luciferase reporter vector (#1471; Promega) between *Kpn*I and *Xho*I restricted enzyme sites. All the reporter vectors were verified by Sanger sequencing. For luciferase activity assays, GCs were harvested after treatment for 24 h, and the *firefly* and *Renilla* luciferase activities were measured using the Dual‐Luciferase Reporter Assay System (#E1910; Promega). The relative luciferase activity of each sample was calculated as the *firefly* activity normalized to *Renilla* activity. Each group has three independent samples and the experiments were performed in triplicates.

### 2.11 Western blotting

After treatment for 48 h, the total protein from porcine GCs was extracted using cold RIPA lysis buffer (#PD0013; Beyotime) with protease inhibitors and 0.1% PMSF (vol/vol). The concentration of each protein sample was quantified through BCA method (#PD0012; Beyotime). Equal amount (~20 μg) of total protein was uploaded and separated by 4%–20% sodium dodecyl sulfate–polyacrylamide gel electrophoresis gels, which was then transferred to polyvinylidene difluoride membranes (Merck Millipore). After blocked with 5% nonfat milk for 1.5 h, the membranes were incubated with primary antibodies at 4°C overnight. After washed with TBST twice for 30 min, the membranes were incubated with species‐specific HRP‐conjugated secondary antibodies for 1 h. The protein blots were visualized and the high‐resolution original images were obtained by a high‐sensitivity ECL detection system (#E412‐01; Vazyme Biotech Co., Ltd.). The densitometry of each blotting image was analyzed by ImageJ software. The primary antibodies were anti‐caspase 3 (#19677‐1‐AP; 1:1000; ProteinTech), anti‐CDK6 (#ab151247; 1:1000; Abcom), anti‐Ki67 (#ab16667; 1:1000; Abcom), anti‐PCNA (#13110; 1:1000; Cell Signaling Technology), anti‐phospho‐SMAD3 (#D155153; 1:1000; Sangon Biotech), anti‐SMAD3 (#sc‐8332; 1:1000; Santa Cruz), and anti‐GAPDH (#198662; 1:3000; Sangon Biotech). The protein level of GAPDH was used as an internal control.

### 2.12 Chromatin immunoprecipitation and ChIP‐qPCR

The enrichment of SMAD4 on the promoter of DElncRNAs under different conditions were detected by chromatin immunoprecipitation (ChIP) and ChIP‐qPCR assays as previously described.^38^ In brief, GCs were collected after treatment for 24–48 h. A total of 1.0 × 10^8^ cells were fixed with 1% formaldehyde for 10 min at 37°C, quenched with 0.15 M glycine for 10 min, and then sonicated to shear DNA to 200–1000 bp length. The SMAD4‐DNA complexes were precleared with Protein A + G agarose and immunoprecipitated with anti‐SMAD4 antibody (#sc‐1909‐R; Santa Cruz) or normal anti‐IgG antibody (#sc‐2358; Santa Cruz). Subsequently, the interacted DNA was isolated and purified using chloroform/phenol method, and further utilized for PCR and qPCR detection. Antibody IgG was used as the internal control and the unprocessed chromatin served as the input control. ChIP‐qPCR signals were calculated as fold enrichment relative to input control signals. SMAD4 ChIP signals were normalized against IgG signals from the same samples. PCR primers used for TEX14‐IT1 promoter enrichment were listed as following: SBE1/3 (280 bp), F: 5′‐TTGCCAGAAGTAAATGGGTAGG‐3′, R: 5′‐TCCCAGGCTAGGAGACAAATC‐3′; SBE‐X (216 bp), used for negative control, F: 5′‐GTCATAGGCTCGCATACAC‐3′, R: 5′‐ACTCCCTTCCCTGCTCAA‐3′.

### 2.13 Cell proliferation assay

For cell proliferation analysis, GCs were seeded into 96‐well culture plates with the density of 1.5 × 10^3^ cells/well for 12 h. After the corresponding treatment, 10 μl Cell Counting Kit‐8 solution (#A311‐01; Vzayme Biotech Co., Ltd.) was added to the culture medium in each well at specific time points (0, 12, 24, 48, and 72 h) after treatment or transfection. After incubation for 1.5 h in 37°C humid atmosphere, the absorbance of cells in each well was measured using a microplate reader with optical density at 450 nm wavelength. Each group has six independent replicates.

### 2.14 Cell apoptosis detection

For cell apoptosis detection, Annexin V‐fluorescein isothiocyanate (FITC)/propidium iodide (PI) double staining apoptosis detection kit (#A211‐01; Vzayme Biotech Co., Ltd.) was performed according to the manufacturer's instruction. Briefly, GCs were harvested after treatment for 48 h and gently washed with cold PBS twice, further stained with 5 μl Annexin V‐FITC and 5 μl PI. Then, 2.0 × 10^5^ cells were detected by fluorescence‐activated cell sorting on a cell counting machine (Becton Dickinson). The apoptosis rate was analyzed using the Flowjo v10.0 software which was considered as the ratio of (cell numbers in Q2 and Q3)/total cell numbers.^39^

### 2.15 Morphometric analysis

After transfection or treatment for 24–48 h, morphometric analysis was conducted to evaluate the effects of TGF‐β1 and TEX14‐IT1 on the morphology of porcine GCs cultured in vitro. Five different detection area from each sample were randomly selected and high‐solution images of porcine GCs were obtained from Odyssey Imaging System (LI‐COR Biosciences). Three independent samples were set in each group.

### 2.16 Statistics analysis

Statistics analysis was performed using GraphPad Prism v7.0 and SPSS v20.0 (SPSS). Data in this study were shown as mean ± SEM with at least three replicates. The significance between two groups was calculated by a two‐tailed unpaired Student's *t*‐test. For three or more groups comparison, analysis of variance was performed followed by S‐N‐K post hoc multiple comparisons. *p < 0.05 and **p < 0.01 were considered as significant and extremely significant, respectively.

## 3 RESULTS

### 3.1 Transcriptome sequencing for lncRNAs identification

**FIGURE 1:**
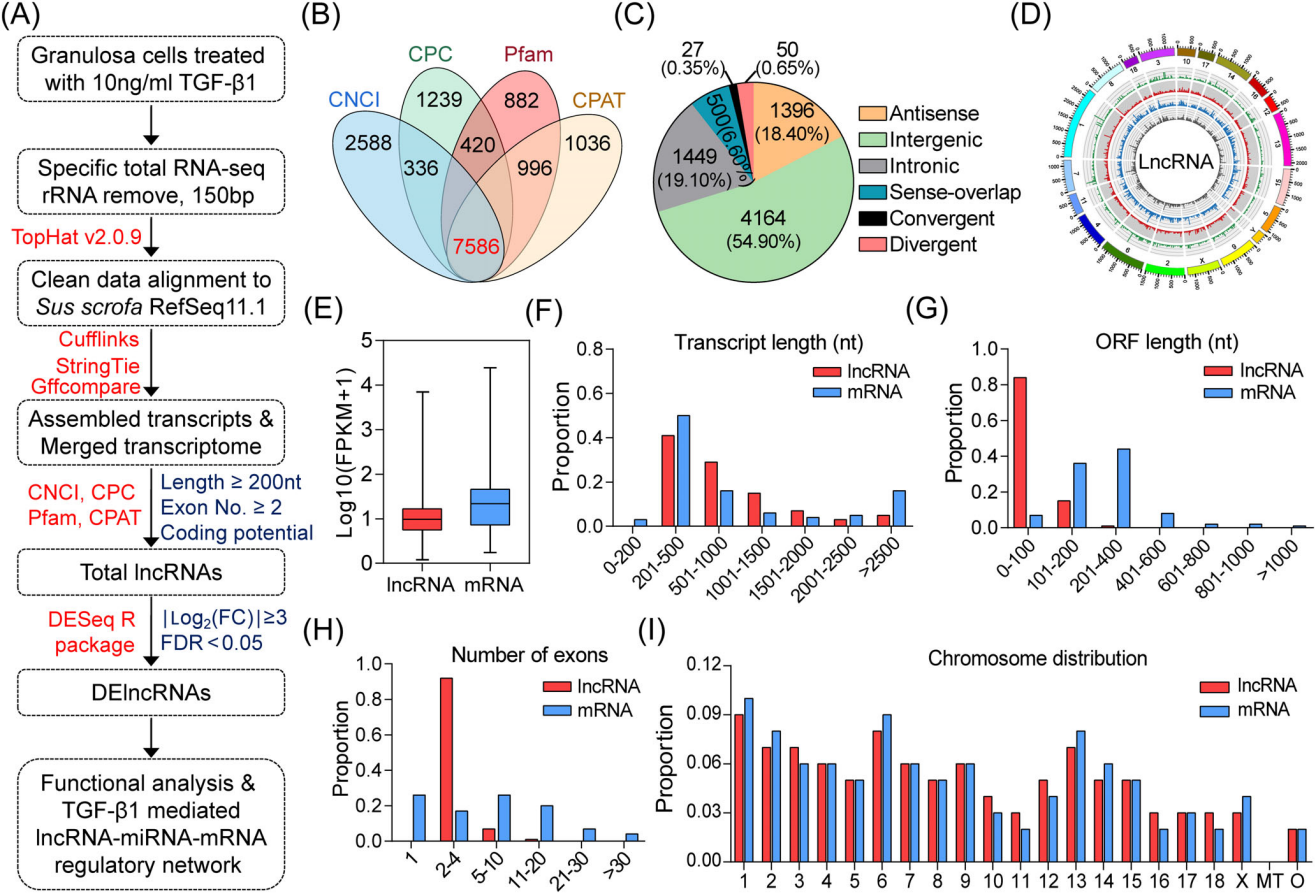
Identification of the lncRNA profiles in porcine GCs treated with TGF‐β1. (A) Flow diagram depicting the strategy for DElncRNAs identification and the lncRNA regulatory network construction using RNA‐seq and bioinformatic analysis. Software used in this study were indicated in red, and the criteria for lncRNAs and DElncRNAs identification were shown in blue. (B) Venn diagram showing the lncRNAs identified by four different algorithms (CNCI, CPC, Pfam, and CPAT). (C) The pie chart showing the number and proportion of lncRNAs classified according to their genomic location. (D) Circos plot showing the chromosome distribution of the total lncRNAs identified in TGF‐β1‐treated porcine GCs. The outermost ring represents different chromosomes and the four inside rings with bars in colors show the different lncRNA types, sense lncRNAs (green), lincRNAs (red), antisense lncRNAs (gray), and intronic lncRNAs (blue). The height of each bar within the rings represents the expression level by RNA‐seq. (E) Boxplots depicting the expression levels of lncRNAs and mRNAs. (F–I) The distribution of transcript length (F), ORF length (G), exon numbers (H), and chromosome location (I) of lncRNAs (red) and mRNAs (blue) were analyzed and compared.

To identify the lncRNA transcriptomic alteration in porcine GCs under the treatment of 10 ng/ml TGF‐β1, a high‐throughput RNA‐sequencing strategy was designed (Figure 1A). In brief, high quality total RNA from control or TGF‐β1‐treated GCs was isolated to detect the expression profiles and potential functions of lncRNAs based on the RNA‐seq data. A total of 36.75 Gb clean data (~71,769,465 reads per sample) with Q30 > 93.92% were obtained after removing adaptor and filtering out low‐quality reads. Approximately 97.37% reads were mapped to the *Sus scrofa* RefSeq 11.1 using a two‐iteration mapping method. With the cut‐off criteria mentioned above, a total of 7586 lncRNAs including 4164 intergenic (54.90%), 1449 intronic (19.20%), 1396 antisense (18.40%), 500 sense‐overlapping (6.60%), 50 divergent (0.65%), and 27 convergent lncRNAs (0.35%) were identified (Figure 1B,C). Notably, the chromosome distribution of lncRNAs in GCs is uneven, long chromosomes (such as 1, 6, and 13) contains more lncRNAs than short chromosomes (such as 11, 12, and 16) (Figure 1D). In comparison to the mRNAs identified in our previous study,^11^ lncRNAs had a lower average transcript abundance, shorter transcript length (200–1000 nt) and ORF length (0–200 nt), as well as fewer exon numbers (≤4) in GCs (Figure 1E–H). Interestingly, we also noticed that the distribution feature of lncRNAs and mRNAs on different chromosomes in GCs were consistent (Figure 1I).

### 3.2 Identification of the TGF‐β1 highly sensitive DElncRNAs

**FIGURE 2:**
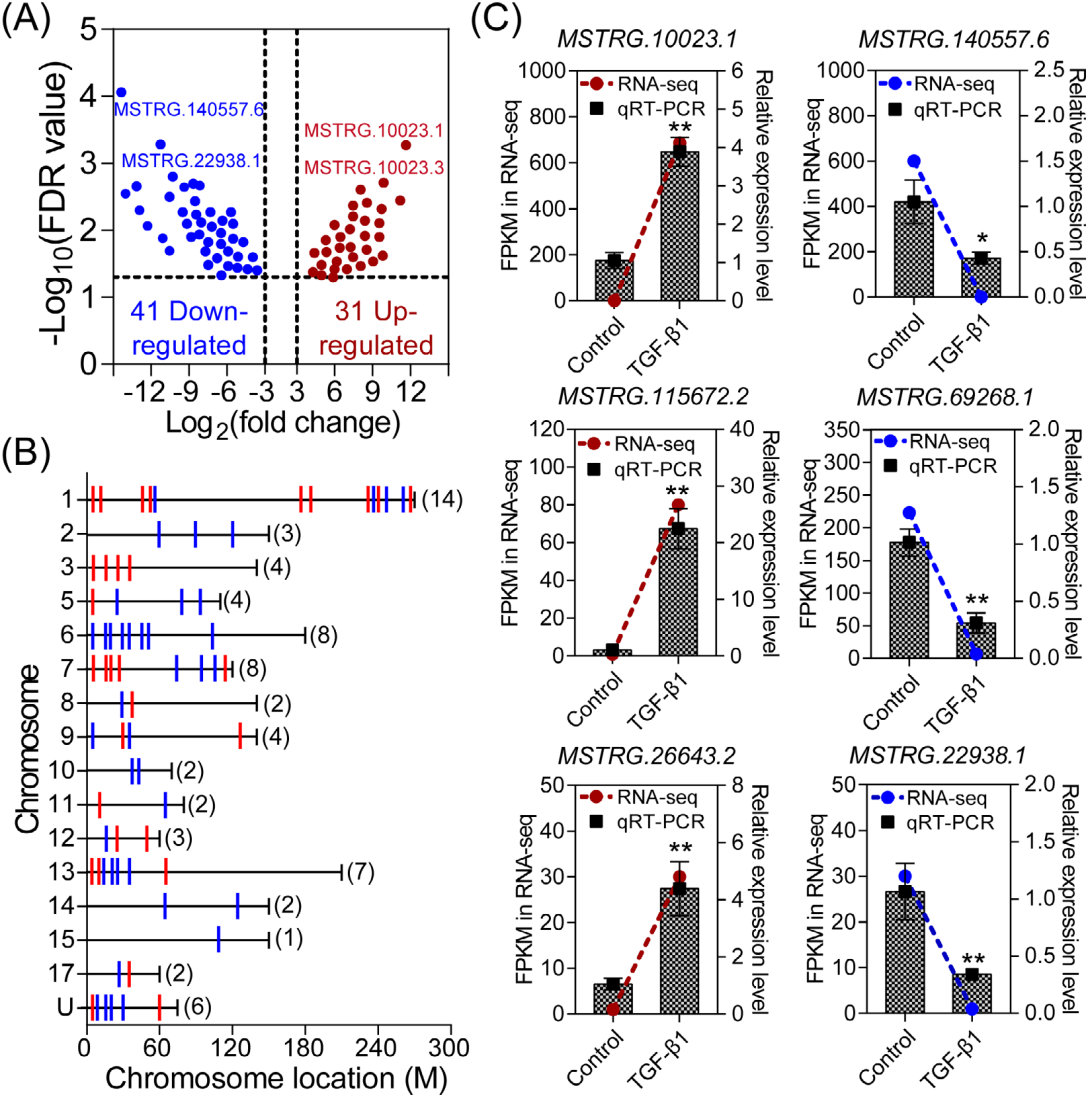
Identification and validation of the DElncRNAs in TGF‐β1‐treated porcine GCs. (A) Volcano plot depicting the profile of DElncRNAs in porcine GCs with the criteria of |log_2_(fold change)| ≥ 3 and adjusted FDR ≤ 0.05. (B) Chromosome distribution and location of the DElncRNAs. Expression patterns of the DElncRNAs were shown in different colors (red indicates upregulation and blue indicates downregulation). The numbers in the brackets depict the amount of DElncRNA within each chromosome. (C) The expression patterns of six randomly selected DElncRNAs in TGF‐β1‐treated porcine GCs were validated by qRT‐PCR (*n* = 3). Data from qRT‐PCR assays were shown as mean ± SEM with three independent replicates. *p* values were calculated by a two‐tailed Student's *t*‐test. *p < 0.05; **p < 0.01.

To gain insight into the crucial lncRNAs in response to TGF‐β1 in GCs, we further analyzed the DElncRNAs and a total of 72 DElncRNAs (31 upregulated and 41 downregulated lncRNAs) were identified with the cut‐off criteria of |log_2_(fold change)| ≥ 3 and adjusted FDR < 0.05 (Figure 2A and Table S3). According to their expression pattern, top 10 upregulated and downregulated DElncRNAs were listed in Table 1. Among which, MSTRG.10023.3 and MSTRG.140557.6 were considered as the most upregulated and downregulated DElncRNAs. Consistent with the total lncRNAs, the distribution of DElncRNAs have a certain chromosome preference, which are mainly enriched on chromosome 1 (*n* = 14), 6 (*n* = 8), 7 (*n* = 8), and 13 (*n* = 7). While, no DElncRNA was found from chromosome 4, 6, 18, and X (Figure 2B). Besides, six DElncRNAs (three upregulated and three downregulated) were randomly selected for qRT‐PCR validation. As shown in Figure 2C, their expression pattern in TGF‐β1‐treated GCs was consistent with the RNA‐seq data, indicating a high accuracy of RNA‐seq data and demonstrating that these DElncRNAs have higher sensitivity to TGF‐β1.

**TABLE 1:** Top 10 upregulated and downregulated DElncRNAs in TGF‐β1‐treated porcine GCs

lncRNA ID	FDR	log_2_(FC)	Regulation	Chr.^a^	Location
MSTRG.10023.1	5.37E−04	11.65	Up	1	179,456,983–182,979,103
MSTRG.139845.3	3.75E−03	10.28	Up	Unknown	38,626–51,024
MSTRG.10023.3	1.95E−03	9.86	Up	1	179,456,983–182,979,098
MSTRG.139845.12	3.11E−03	9.21	Up	Unknown	48,139–51,076
MSTRG.115672.2	3.93E−03	8.89	Up	7	10,867,820–10,868,339
MSTRG.139845.4	5.36E−03	8.45	Up	1	48,130–51,932
MSTRG.139832.6	6.26E−03	8.24	Up	1	69–9630
MSTRG.26643.2	7.25E−03	8.03	Up	12	34,885,721–34,899,646
MSTRG.122822.4	9.27E−03	7.69	Up	7	116,785,963–116,787,153
MSTRG.81341.6	1.04E−02	7.53	Up	3	25,187,249–25,191,913
MSTRG.140557.5	7.69E−03	−8.05	Down	6	11,726–14,637
MSTRG.139833.2	7.10E−03	−8.16	Down	6	111–2859
MSTRG.139832.21	5.87E−03	−8.43	Down	1	8352–9597
MSTRG.139845.14	8.06E−03	−9.17	Down	Unknown	48,143–51,932
MSTRG.139832.25	2.02E−03	−9.94	Down	13	9803–11,407
MSTRG.140557.7	1.93E−03	−10.00	Down	6	11,726–14,637
MSTRG.140557.2	1.91E−03	−10.02	Down	6	11,726–14,637
MSTRG.22938.1	1.58E−03	−10.28	Down	11	66,679,056–66,718,575
MSTRG.139845.11	6.59E−04	−11.52	Down	Unknown	48,139–51,076
MSTRG.140557.6	8.72E−05	−14.37	Down	6	11,726–14,637

Abbreviations: DElncRNA, differentially expressed long noncoding RNA; FC, fold chage; FDR, false discovery rate; GC, granulosa cell.

^a^ Chr. indicates the chromosomes in porcine genome.

### 3.3 Functional assessment of the DElncRNAs

**FIGURE 3:**
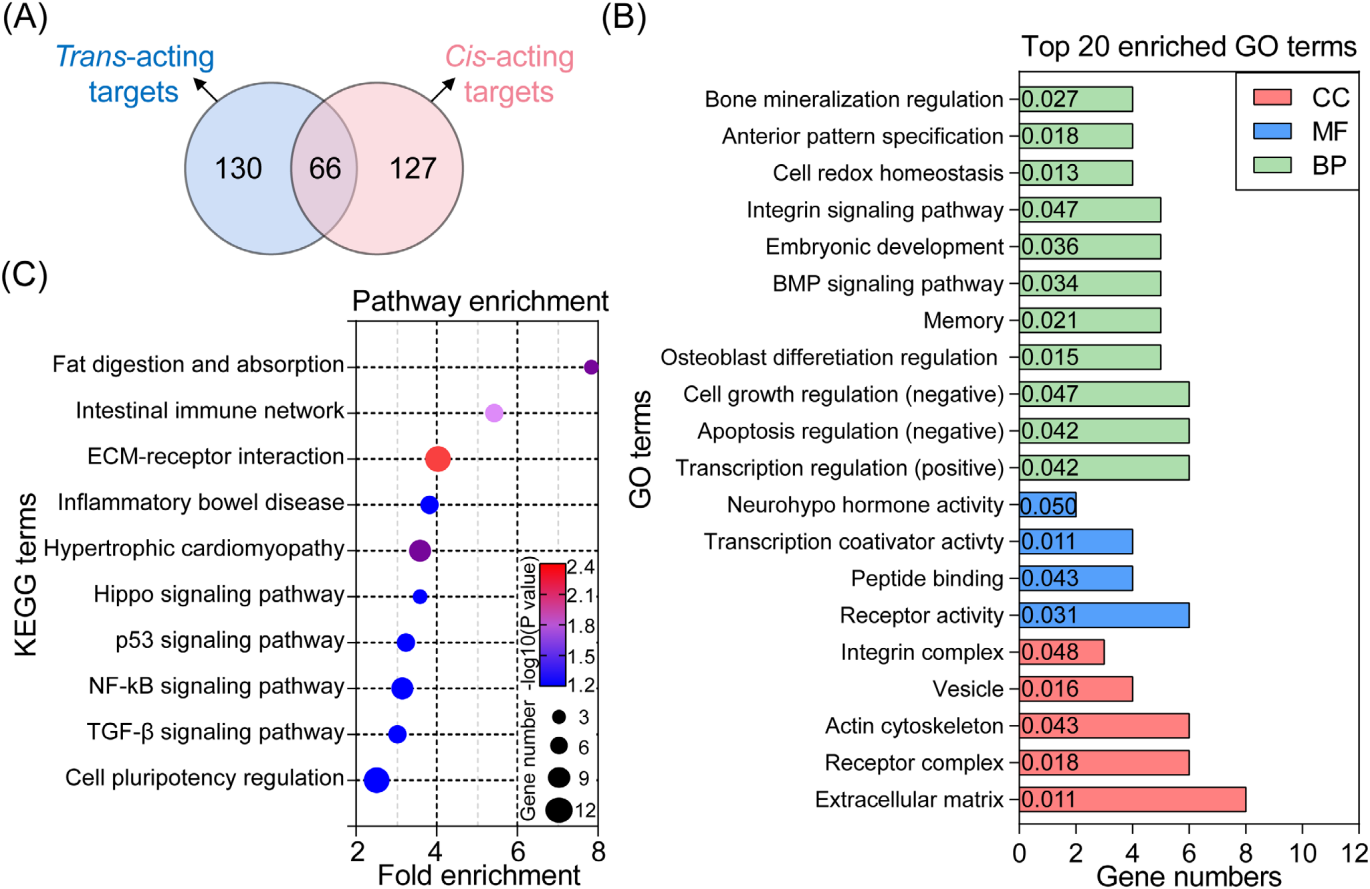
Functional analyses of the DElncRNAs. (A) Venn diagram showing the potential targets of DElncRNAs obtained from both *trans*‐ and *cis*‐regulation modes. (B) GO analyses of the DElncRNAs in porcine GCs treated with TGF‐β1. Top 20 most enriched GO terms were listed. The columns in red, blue, and green indicate the significantly enriched terms of CC, MF, and BP categories. The *p* value of each GO term was also shown in the corresponding column. (C) KEGG pathway enrichment analyses of DElncRNAs and the top 10 significant KEGG terms were presented. The color and size of dots indicates *p* value and gene number, respectively

To evaluate the potential functions of TGF‐β1‐mediated DElncRNAs in GCs, their candidate targets by *trans*‐ and *cis*‐acting regulatory modes were predicted. As shown in Figure 3A, a total of 323 potential targets (196 *trans*‐ and 193 *cis*‐target genes) were identified (Table S4), and 66 common targets were potentially regulated via both *trans*‐ and *cis*‐acting mode. Based on these targets, GO analyses were performed and 34 significantly enriched GO terms were identified (*p* < 0.05), including 25 biological process (BP), 4 molecular function (MF), and 5 cell component (CC) terms (Table S5). As shown in Figure 3B, top 20 significantly enriched GO terms were presented, and “Cell redox homeostasis”, “Receptor activity”, and “Extracellular matrix” were considered as the most enriched GO terms in BP, MF, and CC categories. Besides, we noticed that DElncRNAs were mainly enriched in cell growth, mitosis, and apoptosis‐related GO terms, which is consistent with the functions of TGF‐β1 in porcine GCs.^11^, ^22^ Furthermore, KEGG pathway enrichment analyses were performed and 10 significantly enriched signaling pathways were identified (*p* < 0.05), including TGF‐β, nuclear factor kappa B (NF‐κB), p53, and Hippo signaling pathways (Figure 3C and Table S6). Notably, the significant enriched pathways were mainly involved in the regulation of cell states (proliferation and apoptosis) and functions (response to stimulates). Our findings demonstrate that these DElncRNAs have a wide range of potential functions, which may further mediate the effects of TGF‐β1 in GCs.

### 3.4 Identification of the TGF‐β1‐mediated DElncRNA–DEmRNA interactions

**FIGURE 4:**
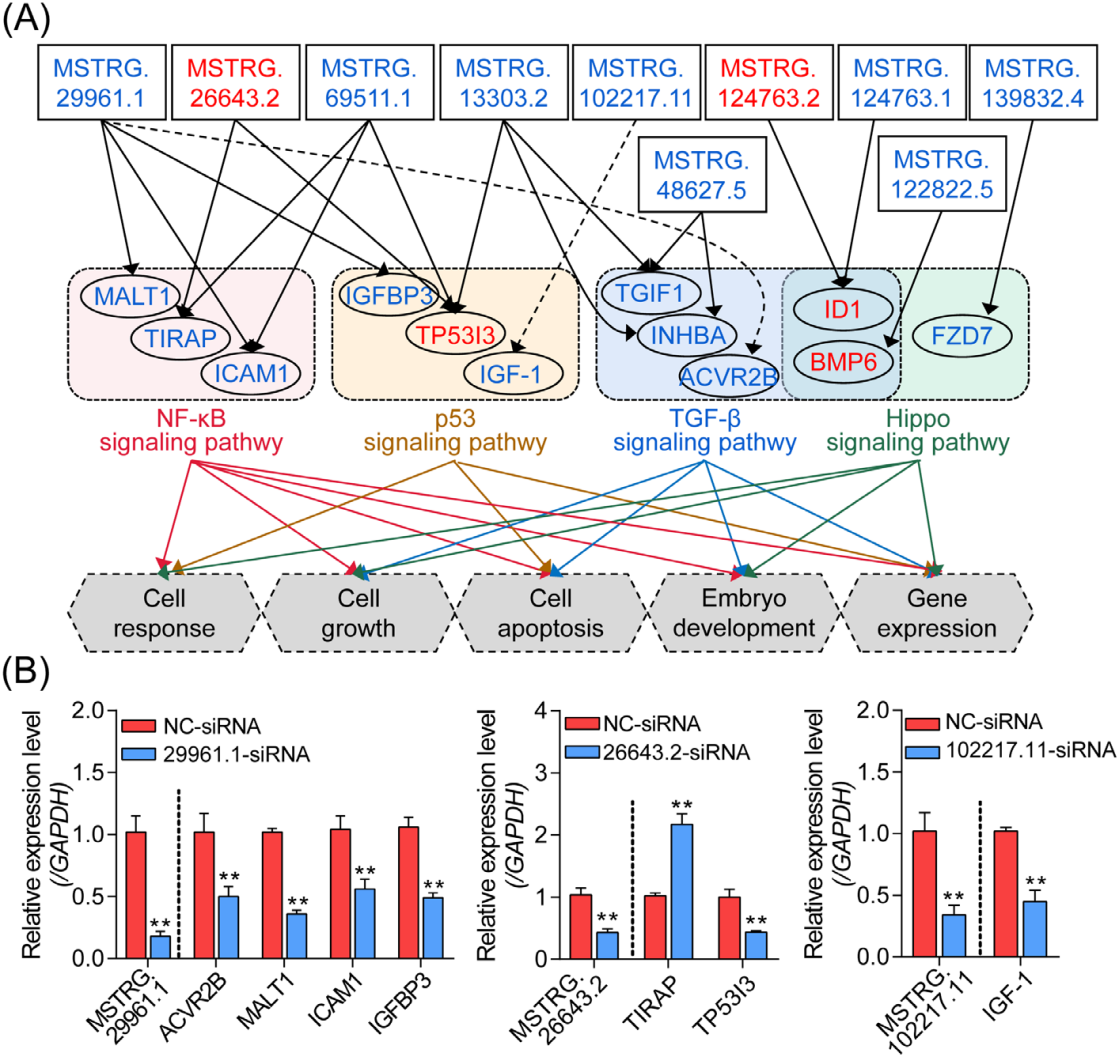
Construction of the TGF‐β1‐mediated DElncRNA–DEmRNA regulatory network. (A) TGF‐β1‐mediated DElncRNA–DEmRNA pathway function network. lncRNAs and mRNAs in the network were indicated by rectangles and ovals. The expression patterns of RNAs are shown in different colors, red indicates upregulation and blue indicates downregulation. The black arrows and dotted black arrows indicate *trans*‐ and *cis*‐regulatory interaction between DElncRNAs and DEmRNAs. Four significantly enriched signaling pathways (NF‐κB, p53, TGF‐β, and Hippo) and their involved functions in porcine GCs were also presented. (B) The interactions within the DElncRNA–DEmRNA regulatory network were validated. The expression levels of corresponding targets in porcine GCs after inhibition of three DElncRNAs were analyzed by qRT‐PCR (*n* = 3). Data from qRT‐PCR were presented as mean ± SEM with three independent replicates. **p < 0.01 by a two‐tailed Student's *t*‐test.

Based on the coexpressed targets, the DElncRNA–DEmRNA regulatory networks were constructed to elucidate the role of DElncRNAs in mediating TGF‐β1 functions. As shown in Figure S1, the regulatory networks were established according to the *trans*‐ and *cis*‐acting mode, respectively. In the *trans*‐regulatory network, 306 interactions were identified between 28 DElncRNAs and 168 DEmRNAs. Meanwhile, 167 interactions between 53 DElncRNAs and 96 DEmiRNAs were existed in the *cis*‐regulatory network. In addition, the DElncRNA–DEmRNA interactions involved in the regulation of cell state and function were analyzed. As shown in Figure 4A, 19 interactions among 10 DElncRNAs and 12 DEmRNAs enriched in multiple crucial pathways and involved in the regulation of GC state, embryo development, and gene expression were identified. Besides, several hub genes including two DElncRNAs (MSTRG.26643.2, MSTRG.29961.1), and five (TP53I3, ICAM1, ID1, and INHBA TGIF1) were noticed with higher interaction degree. To further verify the accuracy of the regulatory network, seven interactions were selected for qRT‐PCR validation (Figures 4B and S2), and results demonstrate that the DElncRNA–DEmRNA regulatory network is authentic, indicating that lncRNAs may partially mediate the regulation of TGF‐β1 to the expression of downstream targets.

### 3.5 DElncRNA‐mediated PPI network construction and module identification

**FIGURE 5:**
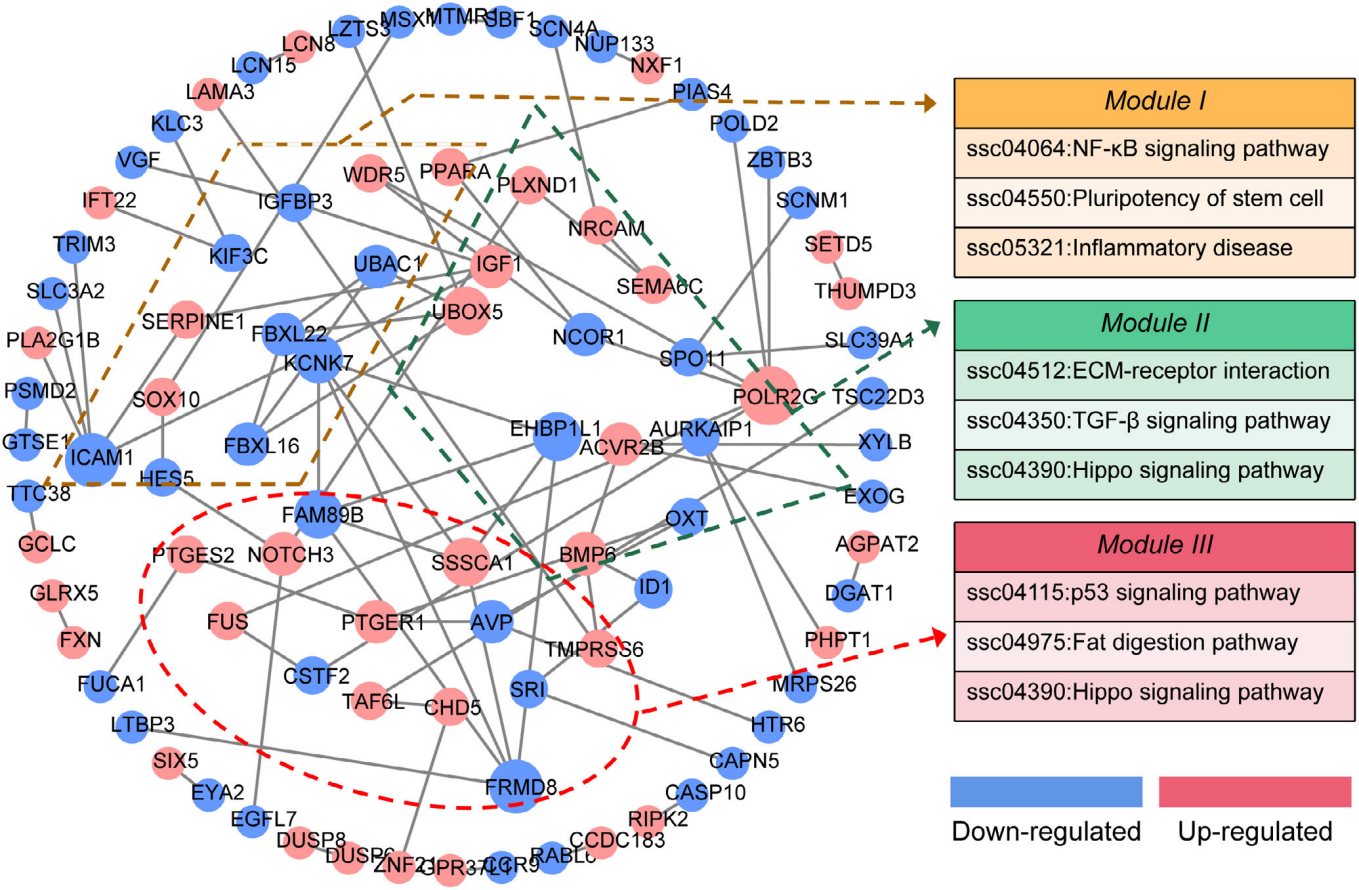
Identification of the DElncRNA‐mediated PPI network. The DElncRNA‐mediated PPI network was established according to the targets of DElncRNAs identified in this study. A total of 90 nodes (protein‐coding targets of DElncRNA) were identified in the PPI network. The nodes in red and blue indicate upregulated and downregulated in TGF‐β1‐treated porcine GCs, respectively. Degrees are shown as the size of the nodes. The dotted areas indicate three significant modules (Module I in orange, Module II in green, and Module III in blue). The most enriched signaling pathways (*p* < 0.05) in each module were presented.

To construct the DElncRNA‐mediated PPI network, 323 protein‐coding targets of DElncRNAs were selected for interaction analysis using the STRING online database and Cytoscape v3.7.2 visualization tool. As shown in Figure 5, 90 nodes (39 upregulated and 51 downregulated) and 103 edges were identified in the DElncRNA‐mediated PPI network. After detection, the enrichment *p* value is less than 1.00E−04, the AND is 1.15, and the ALCC is 0.52, indicating that the network is reliable. Besides, eight high degrees nodes (top 5%, average degree ≥ 5) were considered as hub genes, including *POLR2G*, *ICAM1*, *FRMD8*, *UBOX5*, *KCNK7*, *FAM89B*, *SSSCA1*, and *EHBP1L1*. In addition, three significant enriched modules, termed as Module I, II, and III, were also identified. KEGG analyses showed that the significantly enriched signaling pathways in which the three modules were mainly involved were associated with multiple crucial pathways, including TGF‐β, NF‐κB, p53, Hippo, pluripotency, inflammatory and extracellular matrix‐receptor pathways (Figure 5).

### 3.6 Identification of the DElncRNA–DEmiRNA–DEmRNA competitive endogenous RNA network

**FIGURE 6:**
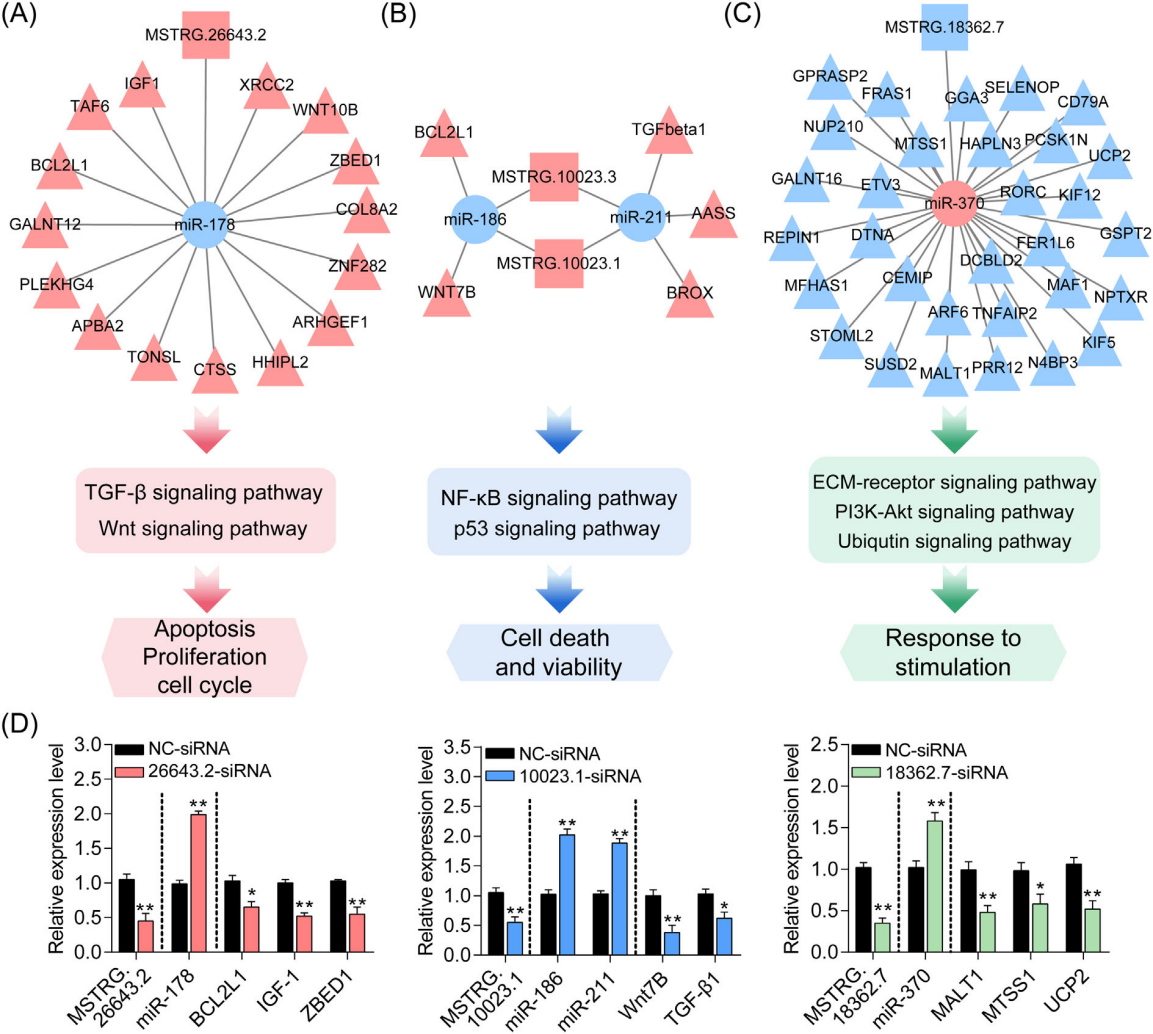
TGF‐β1‐mediated DElncRNA–DEmiRNA–DEmRNA ceRNA regulatory network identification and functional analysis. (A–C) DElncRNA–DEmiRNA–DEmRNA ceRNA networks for apoptosis, proliferation and cell cycle (A), death and viability (B), and response to stimulation (C). The rectangles, circles, and triangles in the networks indicate DElncRNAs, DEmiRNAs, and DEmRNAs. The upregulated and downregulated genes are presented in red and blue, respectively. (D) The expression levels of target DEmiRNAs and DEmRNAs in porcine GCs after DElncRNAs inhibition were detected by qRT‐PCR (*n* = 3). Data from qRT‐PCR assays were shown as mean ± SEM with three independent replicates. *p < 0.05 and **p < 0.01 by a two‐tailed Student's *t*‐test.

Recent studies have shown that lncRNA‐mediated competitive endogenous RNA (ceRNA) networks are essential for multiple life activities, including immunity, embryo implantation, metabolism, and diseases.^40^, ^41^, ^42^, ^43^ To construct the DElncRNA–DEmiRNA–DEmRNA ceRNA regulatory network, the negative interactions among DElncRNAs and previously identified DEmiRNAs were explored. After analysis, 19 negative regulatory interactions among 9 DElncRNAs and 14 target DEmiRNAs were identified using RNAhybridand miRDB (Table S7). Subsequently, the ceRNA network was established after combined with the DEmiRNA–DEmRNA negative interactions.^11^ As shown in Figure S3, 106 interactions among 9 DElncRNAs (4 upregulated and 5 downregulated), 14 DEmiRNAs (8 upregulated and 6 downregulated), and 73 DEmRNAs (27 upregulated and 46 downregulated) were identified. In addition, functional assessment showed that it is associated with the regulation of cell state and function (Figure 6A–C). Among which, several regulators have been shown essential in mammalian ovaries, such as TGF‐β1, IGF‐1, Wnt7B, Wnt10B, CTSS, BCL2L1, miR‐186, and miR‐211. Moreover, the interactions within this ceRNA network were verified in GCs transfected with specific siRNAs for three DElncRNAs (Figure 6D). Together, these results demonstrate that the authentic DElncRNA–DEmiRNA–DEmRNA ceRNA network potentially mediates the functions of TGF‐β1 in GCs.

### 3.7 SMAD4 is essential for the TGF‐β1‐mediated transcriptomic alteration

**FIGURE 7:**
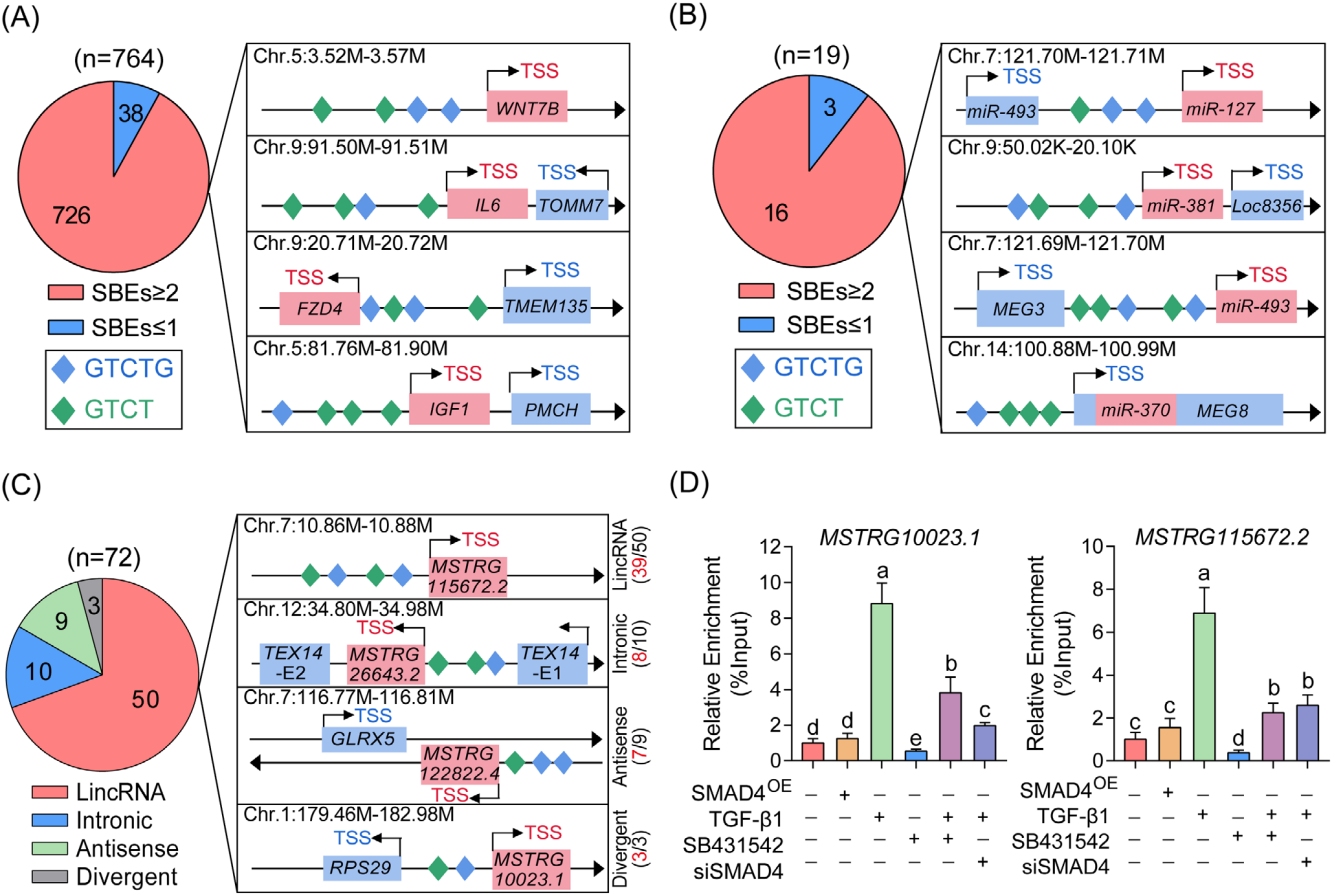
SMAD4 is essential for the TGF‐β1‐mediated transcriptomic alteration. (A–C) The potential SBE motifs within the promoters of TGF‐β1‐induced DEmRNAs (A), DEmiRNAs (B), and DElncRNAs (C) were analyzed. In A and B, pie charts showing the number of genes which contains two and more SBEs (red) or less than two SBEs within their promoters. In C, the pie chart showing the different categories of lncRNAs. (D) The enrichment of SMAD4 on the promoter of *MSTRG10023.1* and *MSTRG.115672.2* in GCs with indicated treatment were detected by ChIP‐qPCR (*n* = 3). Data in D were shown as mean ± SEM with three independent replicates. The different letters labeled in J indicate significant differences.

Dependent on the transcription activity of SMADs is the main approach for canonical TGF‐β pathway to regulate the expression of downstream targets. In our previous study, 890 DEmRNAs (764 of which have accurate genomic sequence) and 19 DEmiRNAs were identified after TGF‐β1 treatment.^11^ Bioinformatics analysis showed that more than 95% DEmRNAs (726/764) and 84% DEmiRNAs (16/19) contain at least two potential SBEs within their promoters (Figure 7A,B). Interestingly, SMAD4 has been proved as TF and mediates the regulation of TGF‐β1 to several DEGs, including *ACVR1B*, *FZD4*, and *TGFBR2*,^3^, ^19^, ^37^ which makes us wonder whether TGF‐β1 alters the lncRNA transcriptome via SMAD4. To address this, the potential SBEs within the promoter of 72 DElncRNAs were analyzed and showed that >75% of which (57/72) contain at least two potential SBEs (Figure 7C). Subsequently, ChIP‐qPCR was performed and results showed that SMAD4 acts as a TF and interacts with the promoter of *MSTRG10023.1* and *MSTRG115672.2* (Figure 7D). Notably, the enrichment of SMAD4 was significantly increased after TGF‐β1 treatment, which was abolished and reversed by SMAD4‐siRNA or 10 μM SB431542. It is also worth noting that SMAD4 overexpression has no effect on the SMAD4 enrichment which could be dramatically reduced by SB431542 treatment. Taken together, these findings demonstrate that the transcription activity of SMAD4 and ligand‐dependent regulatory mechanism are essential and universal for the TGF‐β1‐mediated transcriptomic alteration in GCs, including mRNAs, miRNAs, and lncRNAs.

### 3.8 TGF‐β1 induces *TEX14‐IT1* transcription in a SMAD4‐dependent manner

**FIGURE 8:**
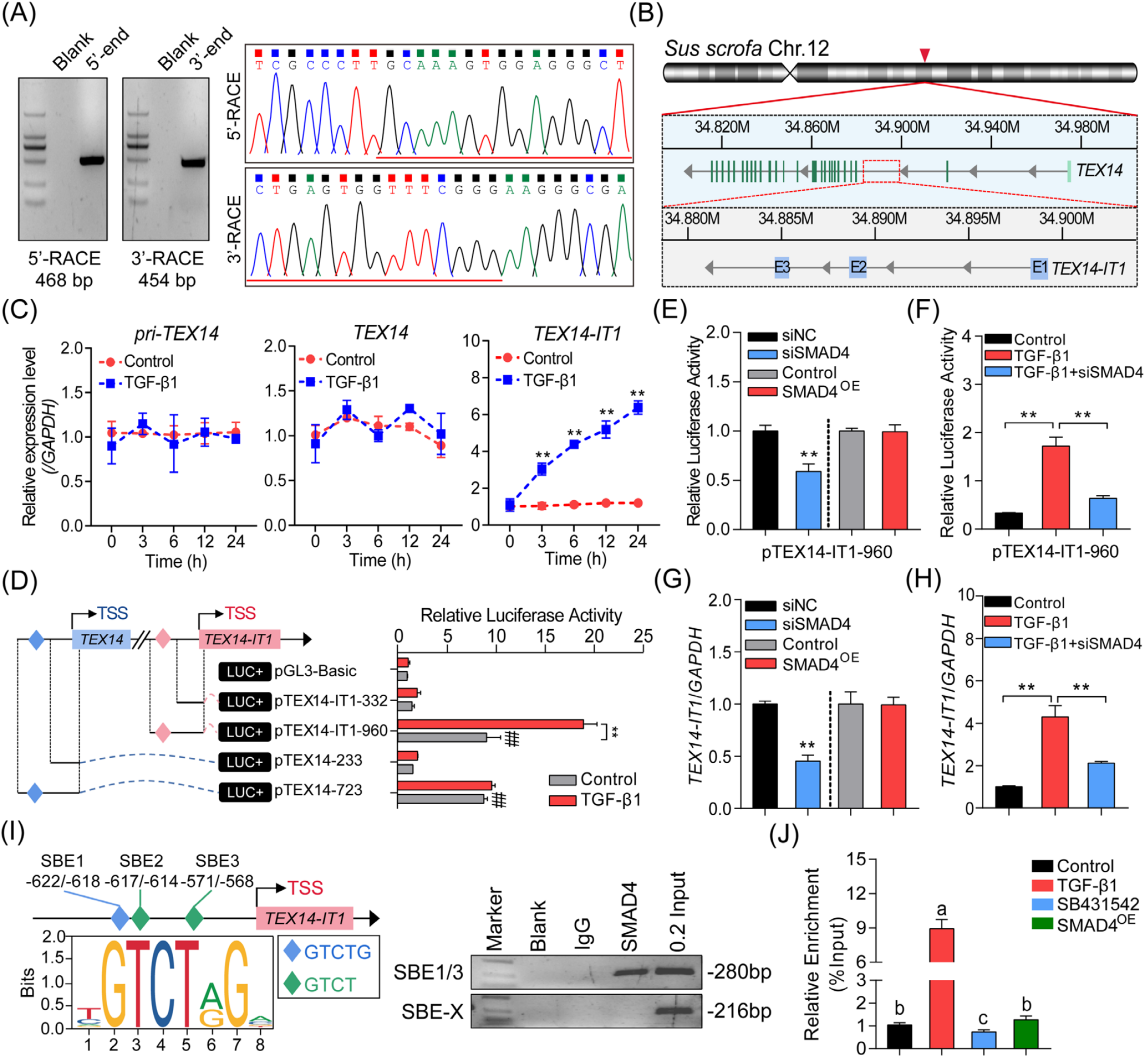
TGF‐β1 induces the transcription of *TEX14‐IT1* in a SMAD4‐dependent manner. (A) Detection of the 5′‐ and 3′‐terminal of TEX14‐IT1 by using RACE. Left panel: gel image. Right panel: Sanger DNA sequencing. (B) Schematic annotation of the genomic locus of *TEX14‐IT1* with associated UCSC Genome Browser. (C) The expression levels of pri‐TEX14, TEX14, and TEX14‐IT1 in porcine GCs treated with 10 ng/ml TGF‐β1 for different time (0, 3, 6, 12, 24 h) were detected by qRT‐PCR (*n* = 3). (D) The effects of TGF‐β1 on the promoter activity of the pig *TEX14* and *TEX14‐IT1* were detected by luciferase activity assays (*n* = 3). The diamonds in blue and pink indicate the potential core promoters of *TEX14* and *TEX14‐IT1*, respectively. (E–H) The effects of TGF‐β1/SMAD4 axis on the promoter activities (E and F) and the expression (G and H) of pig *TEX14‐IT1* were analyzed by luciferase activity assays (*n* = 3) and qRT‐PCR (*n* = 3). (I) Diagram depicting the promoter of *TEX14‐IT1* (left) and ChIP assays (right), the potential SBEs with different motifs within *TEX14‐IT1* promoter were shown as diamonds with different color. SBE‐X in this study serves as a negative control. (J) The enrichment of SMAD4 on the promoter of *TEX14‐IT1* in porcine GCs under different treatment (10 ng/ml TGF‐β1, 10 μM SB431542, and 2.5 μg pcDNA3.1‐SMAD4) were detected by ChIP‐qPCR (*n* = 3). Data were shown as mean ± SEM. **, ^##^ *p* < 0.01. The different letters labeled in J indicate significant differences by one‐way ANOVA analysis.

The above‐mentioned study shows that MSTRG.26643.2, one of the hub gene that highly sensitive to and positively induced by TGF‐β1, is a potential multifunctional lncRNA which is chosen for the following investigation. First, the full‐length sequence of MSTRG.26643.2 was identified by RACE (Figures 8A and S4A). After genome mapping, MSTRG.26643.2 is identified as a 617 nt sense‐intronic lncRNA with 3 exons located within the first intron of *TEX14* (Figure 8B), which was termed as TEX14 intronic transcript‐1, TEX14‐IT1. Characteristic analyses showed that TEX14‐IT1 is a moderately conserved lncRNA evenly distributed in both cytoplasm and nucleus with higher H3K27Ac enrichment around TSS (Figure S4B–D). qRT‐PCR assays showed that TEX14‐IT1, rather than primary TEX14 (pri‐TEX14) or mature TEX14, was significantly induced in GCs treated with TGF‐β1 (Figure 8C). Consistently, the promoter activity of *TEX14‐IT1*, rather than *TEX14*, was also significantly upregulated by TGF‐β1 (Figure 8D), indicating that *TEX14‐IT1* is induced by TGF‐β1 independent of its host gene *TEX14*.

Characteristic analyses showed that the core promoter of *TEX14‐IT1* contains the binding motifs of multiple crucial TFs (Table S8). including three SBEs located at −622/−618, −617/−614, and −571/−568 (Figure S5A), suggesting that SMAD4 mediates the regulation of TGF‐β1 to *TEX14‐IT1*. To address this, luciferase activity assays were performed and showed that knockdown of SMAD4 dramatically inhibited the promoter activity of *TEX14‐IT1* both in the absent and present of TGF‐β1, which was not influenced by SMAD4 overexpression (Figure 8E,F). Notably, data from qRT‐PCR assays were consistent with the observations (Figure 8G,H), indicating that TGF‐β1 activates the transcription of *TEX14‐IT1* in a SMAD4‐dependent manner. In addition, RNA‐seq data and experimental evidence confirmed that SMAD4 influences the transcription of *TEX14‐IT1* independent of its host gene *TEX14* (Figure S5B–D). Furthermore, ChIP assays showed that SMAD4 interacts with the core promoter of *TEX14‐IT1* (Figure 8I), and the enrichment of SMAD4 on the promoter of *TEX14‐IT1* was significantly increased by TGF‐β1, while dramatically decreased by SB431542, but not influenced after SMAD4 overexpression (Figure 8J), demonstrating that SMAD4 mediates the regulation of TGF‐β1 to *TEX14‐IT1* through ligand‐dependent activation mode.

### 3.9 TEX14‐IT1 mediates the antiapoptotic and proproliferative effects of TGF‐β1 in GCs

**FIGURE 9:**
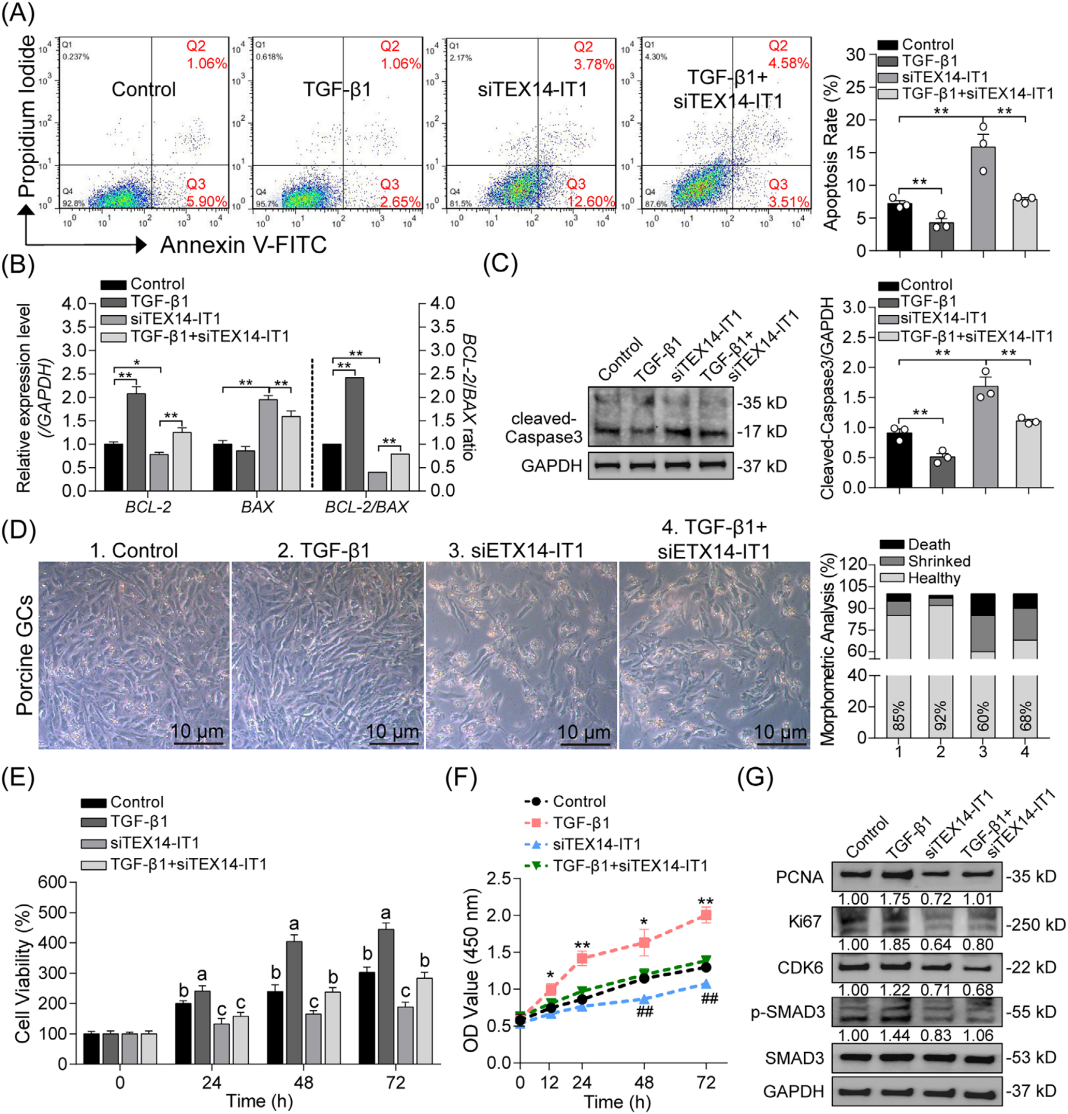
TEX14‐IT1 mediates the antiapoptotic and proproliferative functions of TGF‐β1 in GCs. (A) The apoptosis of porcine GCs under different conditions as indicated was detected by FACS (*n* = 3). (B) The mRNA levels of apoptosis‐related genes (*BCL‐2* and *BAX*) in porcine GCs with indicated treatment were detected by qRT‐PCR assay (*n* = 3), and the ratio of *BCL‐2*/*BAX* was calculated. (C) The protein levels of cleaved‐caspase 3 in porcine GCs with the same treatment mentioned above were measured by western blotting assay (*n* = 3). (D) The morphological features of porcine GCs under indicated treatment were observed and analyzed (*n* = 3). (E) The viability of porcine GCs was detected after treatment for 0–72 h with 24‐h interval (*n* = 6). (F) Porcine GCs were treated or transfected as indicated, and the cell proliferation was detected at the 0, 12, 24, 48, and 72 h time‐point (*n* = 6). (G) The effects of TEX14‐IT1 silencing on the expression levels of proliferation‐related proteins (PCNA, Ki67, and CDK6), as well as p‐SMAD3 and SMAD3 were measured by Western blotting (*n* = 3). Data were shown as mean ± SEM. *p < 0.05 and **p < 0.01. The different letters labeled in E indicate significance between different groups by one‐way ANOVA analysis

We next investigated the effects of TEX14‐IT1 on the roles of TGF‐β1 in porcine GCs, especially the antiapoptotic and proproliferative functions. Flow cytometry analysis showed that knockdown of *TEX14‐IT1* induced porcine GC apoptosis (7.24 ± 0.26 vs. 15.84 ± 1.14, *p* < 0.01), and also impaired the antiapoptotic function of TGF‐β1 (4.29 ± 0.37 vs. 7.84 ± 0.17, *p* < 0.01) (Figure 9A). Consistently, inhibition of *TEX14‐IT1* notably abolished the regulation of TGF‐β1 to the apoptosis‐related genes (*BCL‐2* and *BAX*) and protein (cleaved‐caspase 3) (Figure 9B,C). In addition, morphometric analysis showed that the total cell numbers was dramatically decreased, and the percentage of shrink cells with jagged edges in siTEX14‐IT1 group (~32%) was significantly higher than that in control (15%) or TGF‐β1 group (~8%) (Figure 9D), indicating that TEX14‐IT1 is essential for membrane integrity of GCs. In consistent with the cell morphology, knockdown of *TEX14‐IT1* dramatically reduced the TGF‐β1‐mediated viability of GCs after treatment for 24–72 h (Figure 9E). Furthermore, the effects of TEX14‐IT1 silencing on cell proliferation was detected and showed that loss of TEX14‐IT1 seriously impaired the proproliferative effects of TGF‐β1 in GCs through cell proliferation assay and proliferation‐associated protein detection, such as PCNA, Ki67, and CDK6 (Figure 9F,G). Unexpectedly, we also noticed that knockdown of TEX14‐IT1 could suppress the protein level of p‐SMAD3, the marker for TGF‐β signaling pathway activity, indicating that a feedback regulatory loop exists between TEX14‐IT1 and TGF‐β signaling pathway. Taken together, our findings demonstrate that TGF‐β1 plays antiapoptotic and proproliferative roles in GCs may partially through lncRNAs, such as TEX14‐IT1.

## 4 DISCUSSION

The fate of follicles is determined by the state of GCs, which is regulated by a complicated network consisting of multiple factors. However, it still needs further exploration as there is a lack of in‐depth exploration studies of the holistic omics in the reproductive system.^8^ With the development of high‐throughput sequencing technology, mono‐ and multi‐omics joint analysis, especially RNA‐seq, are widely utilized to identify the crucial target genes and molecules during different BPs.^15^, ^44^ At present, only a few omics studies focused on folliculogenesis,^45^ follicular development,^46^ ovulation,^47^ and differences in the reproductive performance between individuals and breeds.^48^ Although TGF‐β1 is essential for the normal female fertility, its underlying mechanism remains unclear. Recent sequencing studies have demonstrated that abnormal expression of TGF‐β1 leads to significant alterations in the transcriptome of different cell types, and identified various crucial targets, including multiple coding and noncoding genes.^49^, ^50^ Specifically, our previous study provides a comprehensive transcriptomic view in TGF‐β1‐treated GCs which identified 890 DEmRNAs and 19 DEmiRNAs, indicating the irreplaceable role of the miRNA–mRNA network in the regulation of TGF‐β1 to the normal state and function of GCs.^11^ To further identify the functional lncRNAs in TGF‐β1‐mediated inhibition of follicular atresia, we have investigated the lncRNA transcriptome and identified 72 DElncRNAs highly sensitive to TGF‐β1. Functional analyses showed that these DElncRNAs potentially influence the normal state and function of GCs, which is consistent with the roles of TGF‐β1. Taken together, these findings suggest that lncRNAs are crucial for the functional integrity of TGF‐β1, especially in mammalian GCs.

LncRNAs are defined as a class of RNA with transcript length of more than 200 nucleotides and lacking of long ORF or protein‐coding ability.^51^ After miRNAs, lncRNAs rapidly became as a hotspot and reported association with multiple crucial BPs in development and disease, such as proliferation and growth, apoptosis, differentiation, and even carcinogenesis.^52^, ^53^, ^54^ With the improvement of high‐throughput RNA‐seq strategies and bioinformatics analyses, thousands of lncRNAs have been identified to be actively transcribed from the human genome and other organisms, only a few lncRNAs were screened and characterized in domestic animals.^55^ Unlike miRNAs which control female fertility by targeting multiple crucial ovarian function‐related genes,^22^, ^56^ little is known about the role of lncRNAs in the regulation of ovarian function and female fertility. Specifically, in domestic animals such as pigs and sheep, lncRNAs are involved in the steroidogenesis, follicular atresia, and reproductive fecundity differences.^38^, ^45^, ^47^ However, the mechanism remains largely unknown. In addition, compared to the protein‐coding RNAs, lncRNAs have the features of low conserved, unstable, and expressed in a species‐ and tissue‐specific manner,^57^ which made them more suitable as the markers for female fertility and reproductive performance.

The functions and action mechanisms of ncRNAs are mainly determined by their subcellular localization.^58^ For instance, miRNAs, mainly located in the cytoplasm, induced the degradation or inhibited the translation of target mRNAs at posttranscriptional level.^59^ While, recent studies showed that miRNAs in the nucleus mainly activate the transcription of target genes as small activating RNAs.^60^ Consistent with miRNAs, lncRNAs locate either in the cytoplasm or nucleus, but their action mechanism are more complicated and extremely dependent on their subcellular localization. Specifically, lncRNAs retained in the nucleus mainly function by controlling the transcription of target genes in a *cis*‐ or *trans*‐mode via interacting with transcriptional regulatory proteins, chromatin‐modifying factors, and nuclear complexes.^61^, ^62^ Meanwhile, lncRNAs in the cytoplasm are known to influence the stability of target mRNAs and proteins by scaffolding/decoying cytoplasmic regulatory complexes or acting as ceRNAs through pairing with other RNA molecules.^46^ In addition, cytoplasmic lncRNAs encoding small polypeptides are also essential for the cell fate determination.^63^ The ceRNA mechanism, which is the earliest strategy of lncRNAs, has a great impact on the follicular development and reproductive health, even in domestic animals, such as the NORFA/miR‐126 axis in porcine GCs^38^ and the FDNCR/miR‐543 axis in Hu sheep ovaries.^48^ In this study, the TGF‐β1‐mediated lncRNA–miRNA–mRNA ceRNA regulatory networks in GCs were established, and several hub lncRNAs (TEX14‐IT1 et al.) and candidate genes (*NCOA1*, *TGF‐β1*, *TGFBR2*, and *INHBA*) for sow reproductive traits were included, implying that TGF‐β1 maintains normal follicular development and female fertility potentially through the ceRNA regulatory network.

Similar to the protein‐coding genes, the transcription of lncRNAs is initiated by DNA polymerase II and influenced by multiple crucial signaling pathways (TGF‐β, Wnt, p53, NF‐κB, and MAPK) and transcription factors (NFIX, SMADs, SOX2, STAT3, and ZEB1/2) under physiological and pathological conditions.^64^, ^65^ In this study, we have investigated the regulatory mechanism of TGF‐β1 on *TEX14‐IT1*, an independently expressed novel DElncRNA located within the first intron of *TEX14*, and found that TGF‐β1 induced its transcription in a SMAD4‐dependent manner. TGF‐β1, a well‐established central regulator in mammals, regulates the expression levels of lncRNAs at different levels via canonical and noncanonical pathways, which further mediate the functions of TGF‐β1. For instance, several lncRNAs, such as lnc‐TSI, H19X, and Erbb4‐IR, are induced by the canonical TGF‐β1–SMAD3/4 pathway during tissue fibrosis, which further feedback influences the activity of the TGF‐β signaling pathway by inducing R‐SMAD dephosphorylation and I‐SMAD.^66^, ^67^, ^68^ Notably, the promoters of 72 DElncRNAs identified in this study were characterized, and more than 75% (57/72) contained at least two potential SBEs, indicating that the transcription activity of SMAD4 and ligand‐dependent mechanism are necessary and universal for the lncRNA transcriptomic alterations induced by the canonical TGF‐β signaling pathway in GCs. It is also worth noting that, lncRNAs, such as TEX14‐IT1, could feedback mediate the normal functions of TGF‐β1, which was consistent with previous studies,^69^, ^70^ suggesting that lncRNAs are crucial for the functional integrity of TGF‐β1. However, one issue still not fully understood is the accurate manipulation mechanism of the TGF‐β/SMADs pathway to the expression patterns (upregulation or downregulation) of downstream targets, and the regulatory mechanism of TGF‐β1 on the transcription of DElncRNAs without SBE still remains unclear.

In summary, this study revealed the TGF‐β1‐mediated lncRNA profile in GCs, further demonstrated that the transcription activity of SMAD4 is essential for the regulation of lncRNA transcriptome by TGF‐β1. In vitro functional analyses confirmed that lncRNAs, that is, TEX14‐IT1, are critical for the integrity of the biological functions of TGF‐β1, including proproliferative and antiapoptotic effects. Our findings provided new insights into the roles of lncRNAs in TGF‐β1‐mediated follicular fate determination, and identified crucial lncRNA targets for alleviating follicular atresia and maintaining normal female fertility through TGF‐β1.

## AUTHOR CONTRIBUTIONS

**Qiqi Li:** Conceptualization; methodology; formal analysis; writing – reivew and editing; funding acquisition. **Yanan Huo:** Methodology; data curation; assistance. **Siqi Wang:** Methodology; resources. **Liu Yang:** Resources; assistance; data curation. **Qifa Li:** Conceptualization; resources; supervision; project administration; funding acquisition. **Xing Du:** Conceptualization; methodology; formal analysis; writing – reivew and editing; supervision; project administration; funding acquisition. All the authors have reviewed and approved the submitted version.

## CONFLICT OF INTEREST

The authors declare no conflict of interest.

## Supporting information


**FIGURE S1** Construction of the TGF‐β1‐mediated DElncRNA–DEmRNA regulatory network. (A, B) DElncRNA–DEmRNA regulatory networks based on *trans*‐acting (A) and *cis*‐acting (B) regulation mode, respectively. The rectangles and triangles in the networks indicate lncRNAs and mRNAs. The up‐ and downregulated genes are presented in red and blue, respectively.
**FIGURE S2** Knockdown efficiency detection. (A‐E) Identification of the inhibition efficiency of different siRNAs that specifically targeting MSTRG.29961.1 (A), MSTRG.26643.2 (B), MSTRG.102217.11 (C), MSTRG.10023.1 (D), and MSTRG.18362.7 (E) by qRT‐PCR (*n* = 3). The siRNAs labeled in red font were chosen for the following research. Data were shown as mean ± SEM with three independent replicates. *P* values were calculated by an unpaired Student's *t*‐test, *P < 0.05 and **P < 0.01.
**FIGURE S3** Identification of the TGF‐β1‐mediated DElncRNA–DEmiRNA–DEmRNA ceRNA network in porcine GCs. The rectangles, circles, and triangles in the ceRNA networks indicate DElncRNA, DEmiRNAs, and DEmRNAs, respectively. The up‐ and downregulated genes are presented in red and blue. The size of nodes indicate the corresponding degrees in the network.
**FIGURE S4** Identification and characterization of the pig TEX14‐IT1. (A) The full‐length sequence of pig TEX14‐IT1 was identified by RACE assays. Three exons of TEX14‐IT1 were shown in different colors (exon1, red; exon 2, black; exon 3, blue). (B) Schematic annotation of *TEX14‐IT1* with associated UCSC Genome Browser tracks depicting genomic locus, H3K27Ac modification, and mammalian conservation. (C, D) The subcellular location of TEX14‐IT1 in porcine GCs was predicted by Lnclocator 2.0 (C) and verified by nucleus‐cytoplasm isolation (D). The expression levels of *U6* and *GAPDH* were used as the markers for nucleus and cytoplasm, respectively.
**FIGURE S5** SMAD4 has no effect on the transcription of *TEX14*, the host gene of *TEX14‐IT1*. (A) Identification and characterization of the core promoter of pig *TEX14‐IT1*. The binding motifs of crucial TFs within the core promoter of *TEX14‐IT1* were indicated underlined. The SBE1 with “GTCTG” motif was shown in red and SBE2/3 with “GTCT” motifs were indicated in green. (B) The effects of SMAD4 on the promoter activity of pig *TEX14* were detected by luciferase activity assays (*n* = 3). (C) The levels of *TEX14* transcript (FPKM) in porcine GCs after knockdown of SMAD4 according to the RNA‐seq data. (D) The effects of SMAD4 on the expression levels of *pri‐TEX14* and *TEX14* in porcine GCs were detected by qRT‐PCR assays (*n* = 3). Data in B and D were shown as mean ± SEM with three independent replications.
**TABLE S1**. The oligonucleotides used in this study.
**TABLE S2**. The primers used for qRT‐PCR assays
**TABLE S3**. The DElncRNAs in TGF‐β1‐treated porcine GCs
**TABLE S4**. The targets of DElncRNA in porcine GCs treated with TGF‐β1
**TABLE S5**. GO terms that DElncRNAs were significantly enriched in
**TABLE S6**. KEGG pathway analysis of DElncRNAs after TGF‐β1 treatment
**TABLE S7**. The negative interactions between DElncRNAs and DEmiRNA in porcine GCs
**TABLE S8**. The binding motifs of potential TFs within the core promoter of pig *TEX14‐IT1*

## Data Availability

The datasets referred to in this study have been deposited in NCBI Sequence Read Archive database under Accession SUB7402584, Bioproject PRJNA632987.
