# Supplementary material for: TGF‐β1 regulates the lncRNA transcriptome of ovarian granulosa cells in a transcription activity‐dependent manner
Source: Cell Prolif. 2022 Sep 20;56(1):e13336. doi: 10.1111/cpr.13336 (PMC9816938; doi:10.1111/cpr.13336)
Supplement: Supplementary file 1 — FIGURE S1 Construction of the TGF‐β1‐mediated DElncRNA–DEmRNA regulatory network. (A, B) DElncRNA–DEmRNA regulatory networks based on trans‐acting (A) and cis‐acting (B) regulation mode, respectively. The rectangles and triangles in the networks indicate lncRNAs and mRNAs. The up‐ and downregulated genes are presented in red and blue, respectively. FIGURE S2 Knockdown efficiency detection. (A‐E) Identification of the inhibition efficiency of different siRNAs that specifically targeting MSTRG.29961.1 (A), MSTRG.26643.2 (B), MSTRG.102217.11 (C), MSTRG.10023.1 (D), and MSTRG.18362.7 (E) by qRT‐PCR (n = 3). The siRNAs labeled in red font were chosen for the following research. Data were shown as mean ± SEM with three independent replicates. P values were calculated by an unpaired Student's t‐test, *P < 0.05 and **P < 0.01. FIGURE S3 Identification of the TGF‐β1‐mediated DElncRNA–DEmiRNA–DEmRNA ceRNA network in porcine GCs. The rectangles, circles, and triangles in the ceRNA networks indicate DElncRNA, DEmiRNAs, and DEmRNAs, respectively. The up‐ and downregulated genes are presented in red and blue. The size of nodes indicate the corresponding degrees in the network. FIGURE S4 Identification and characterization of the pig TEX14‐IT1. (A) The full‐length sequence of pig TEX14‐IT1 was identified by RACE assays. Three exons of TEX14‐IT1 were shown in different colors (exon1, red; exon 2, black; exon 3, blue). (B) Schematic annotation of TEX14‐IT1 with associated UCSC Genome Browser tracks depicting genomic locus, H3K27Ac modification, and mammalian conservation. (C, D) The subcellular location of TEX14‐IT1 in porcine GCs was predicted by Lnclocator 2.0 (C) and verified by nucleus‐cytoplasm isolation (D). The expression levels of U6 and GAPDH were used as the markers for nucleus and cytoplasm, respectively. FIGURE S5 SMAD4 has no effect on the transcription of TEX14, the host gene of TEX14‐IT1. (A) Identification and characterization of the core promoter of pig TEX14‐IT1. T [file CPR-56-e13336-s001.docx]

**
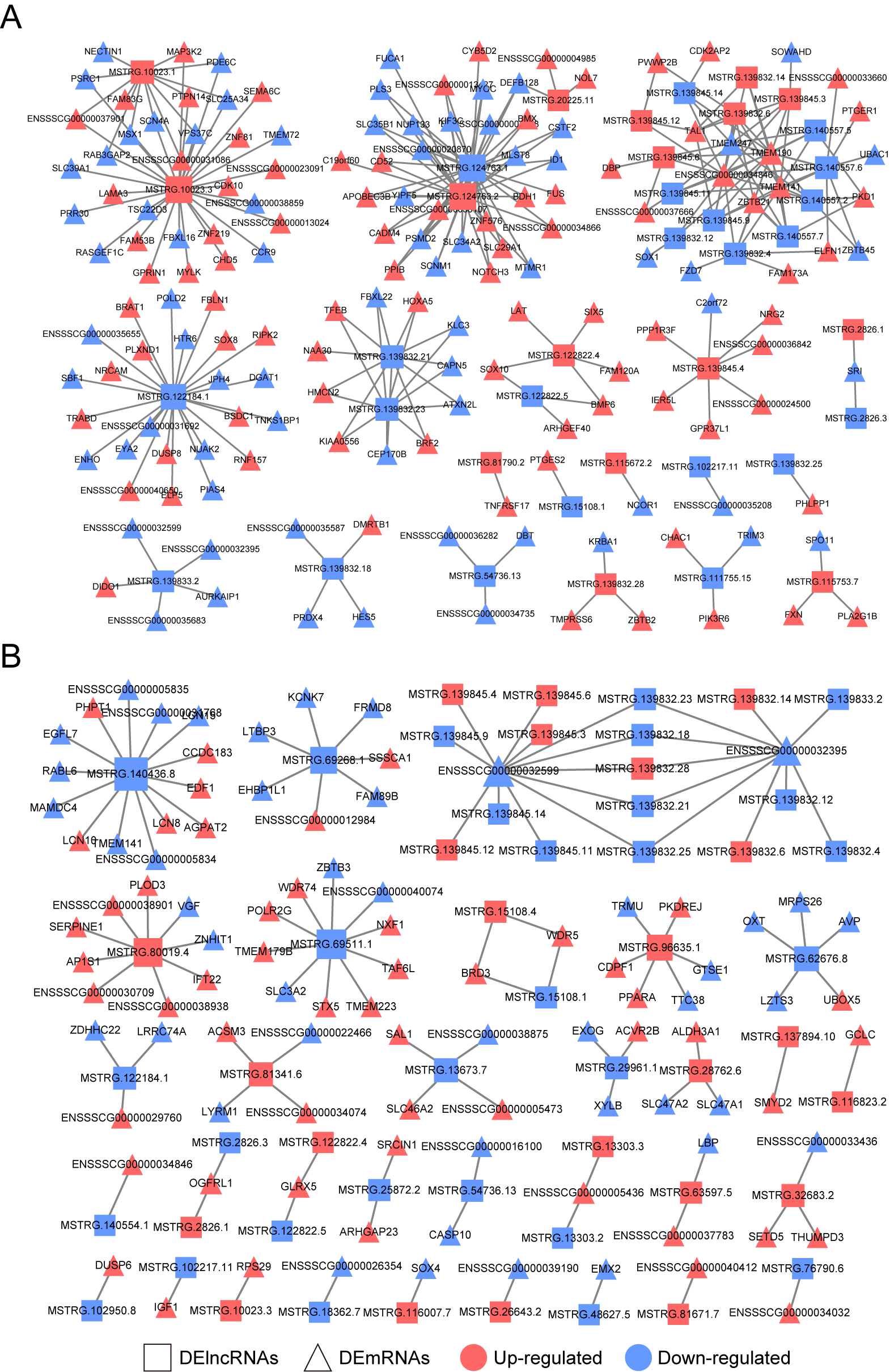
**

**FIGURE S1** Construction of the TGF-β1-mediated DElncRNA-DEmRNA regulatory network. (A, B) DElncRNA-DEmRNA regulatory networks based on *trans*-acting (A) and cis-acting (B) regulation mode, respectively. The rectangles and triangles in the networks indicate lncRNAs and mRNAs. The up- and down-regulated genes are presented in red and blue, respectively.

**
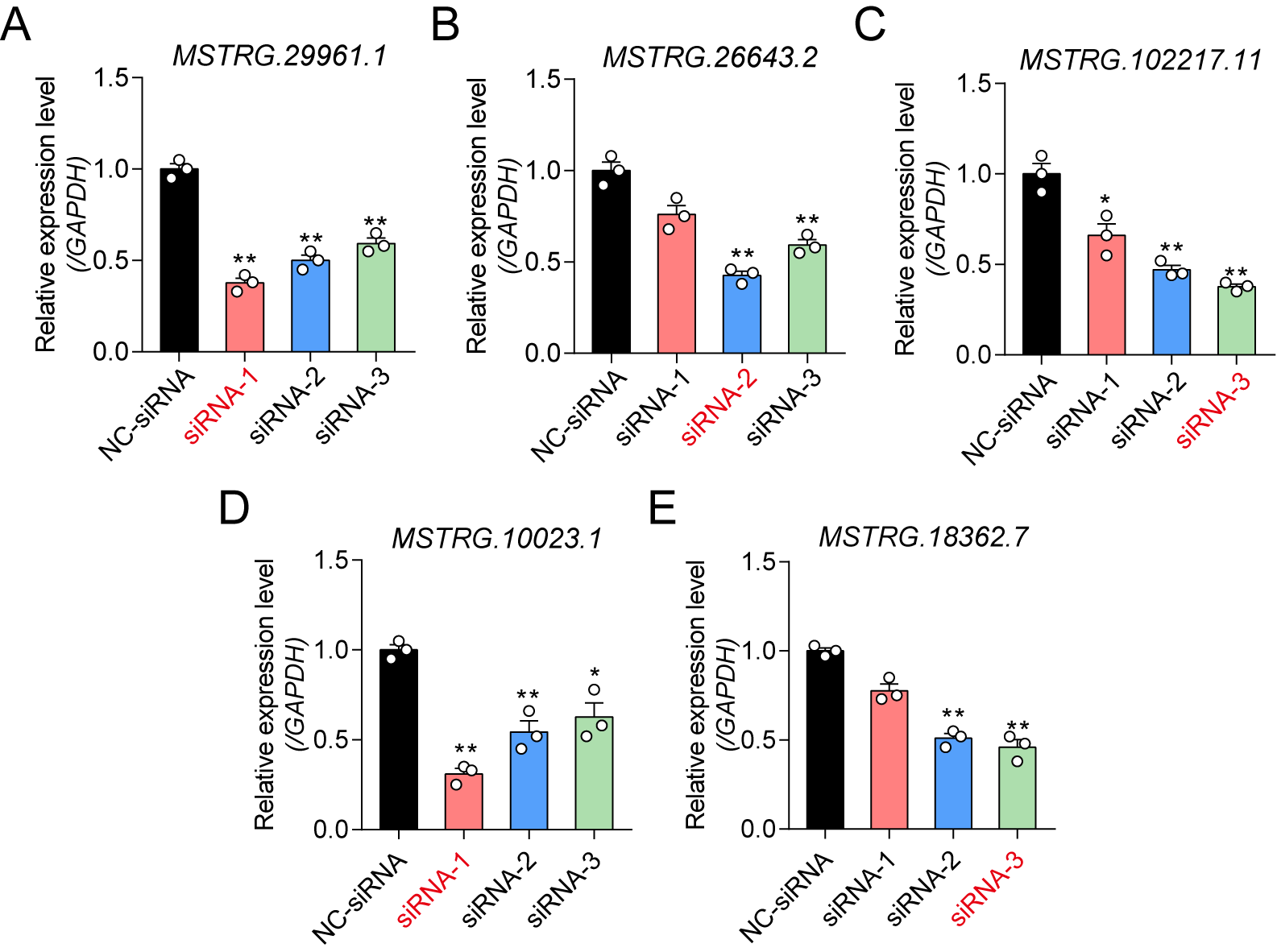
**

**FIGURE S2** Knockdown efficiency detection. (A-E) Identification of the inhibition efficiency of different siRNAs that specifically targeting MSTRG.29961.1 (A), MSTRG.26643.2 (B), MSTRG.102217.11 (C), MSTRG.10023.1 (D), and MSTRG.18362.7 (E) by qRT-PCR (*n*=3). The siRNAs labeled in red font were chosen for the following research. Data were shown as mean ± SEM with three independent replicates. *P* values were calculated by an unpaired Student’s *t*-test, **P*<0.05 and ***P*<0.01.

**
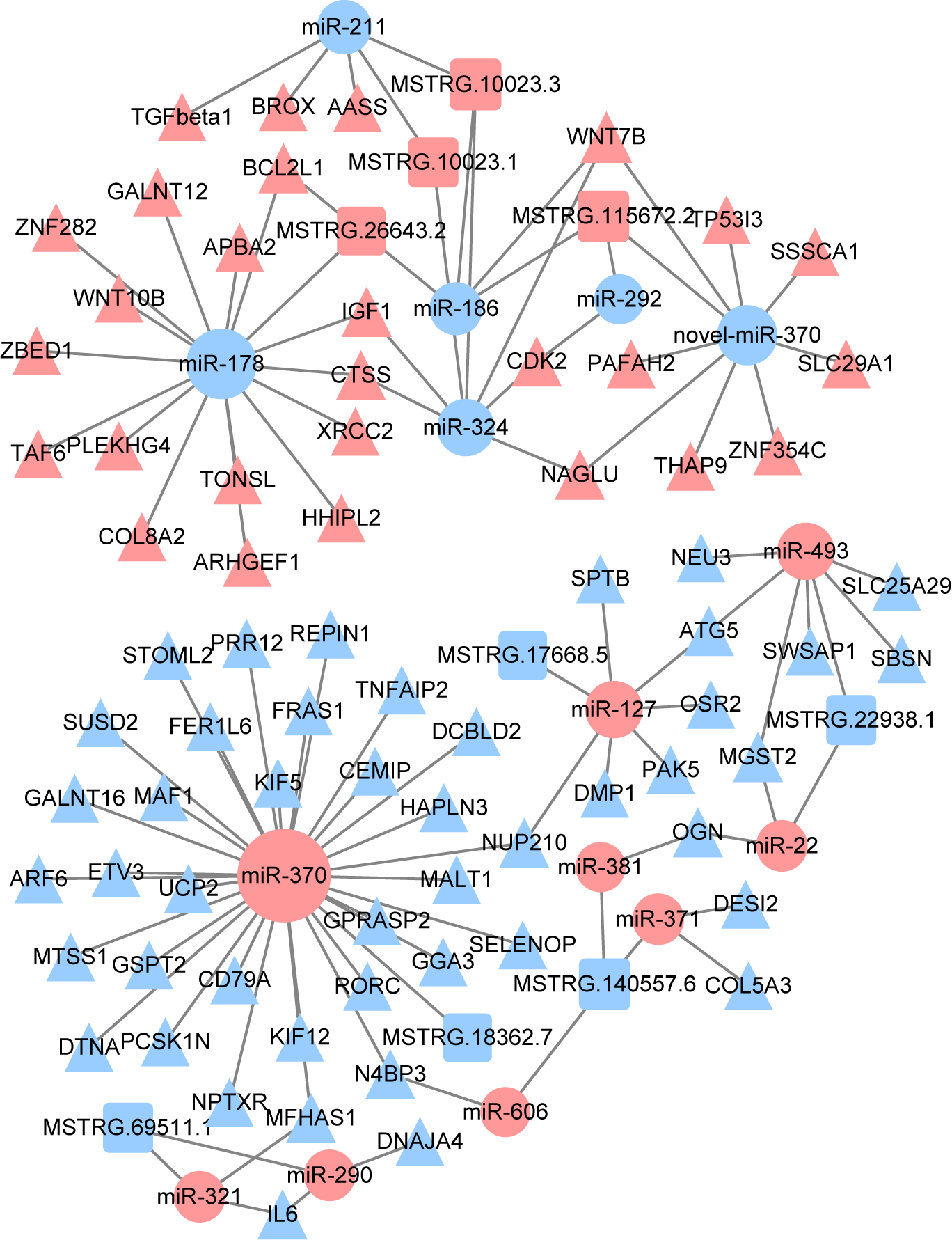
**

**FIGURE S3** Identification of the TGF-β1-mediated DElncRNA-DEmiRNA-DEmRNA ceRNA network in porcine GCs. The rectangles, circles, and triangles in the ceRNA networks indicate DElncRNA, DEmiRNAs, and DEmRNAs, respectively. The up- and down-regulated genes are presented in red and blue. The size of nodes indicate the corresponding degrees in the network.


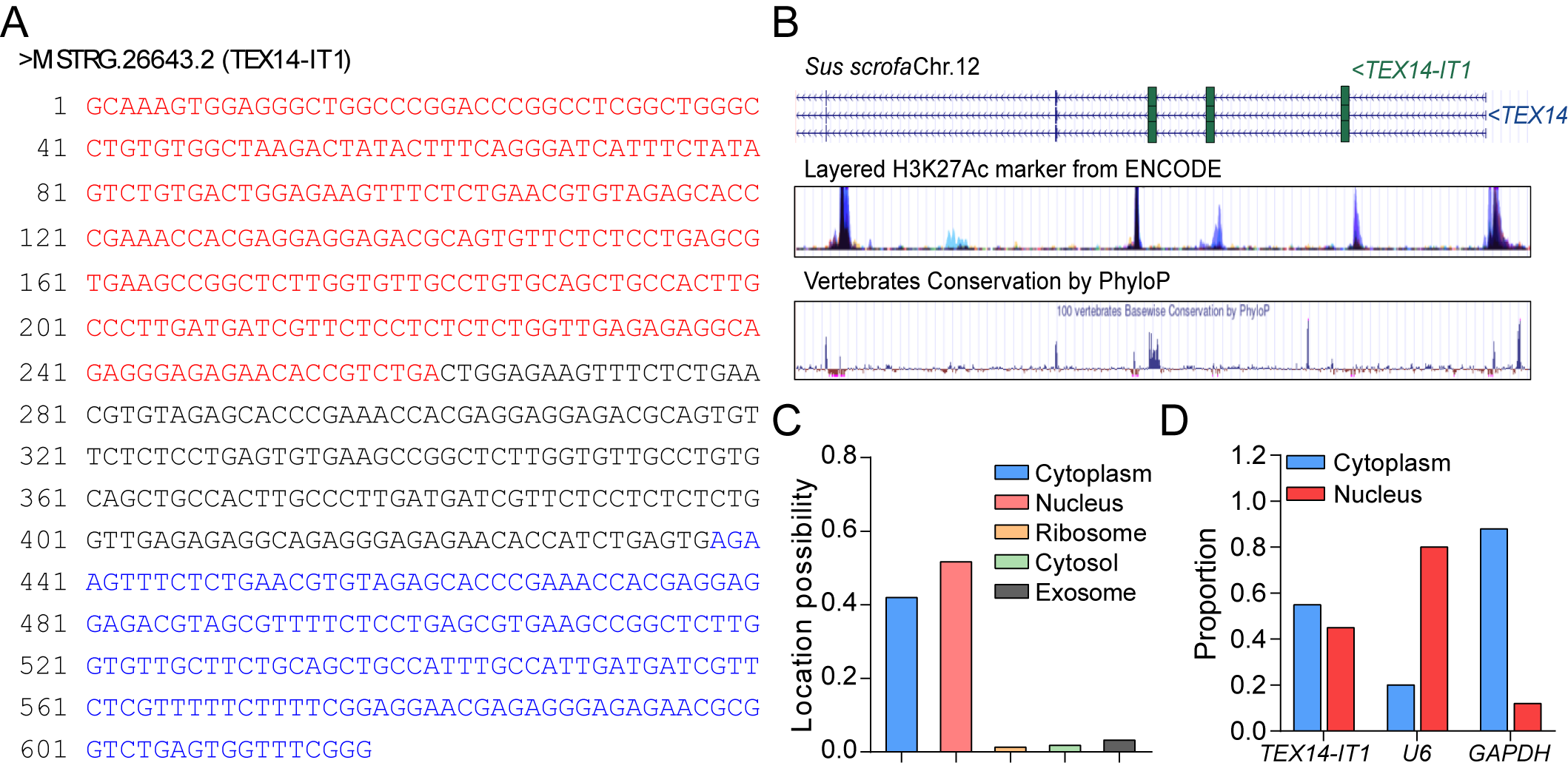


**FIGURE S4** Identification and characterization of the pig TEX14-IT1. (A) The full-length sequence of pig TEX14-IT1 was identified by RACE assays. Three exons of TEX14-IT1 were shown in different colors (exon1, red; exon 2, black; exon 3, blue). (B) Schematic annotation of *TEX14-IT1* with associated UCSC Genome Browser tracks depicting genomic locus, H3K27Ac modification, and mammalian conservation. (C, D) The subcellular location of TEX14-IT1 in porcine GCs was predicted by Lnclocator 2.0 (C) and verified by nucleus-cytoplasm isolation (D). The expression levels of *U6* and *GAPDH* were used as the markers for nucleus and cytoplasm, respectively.


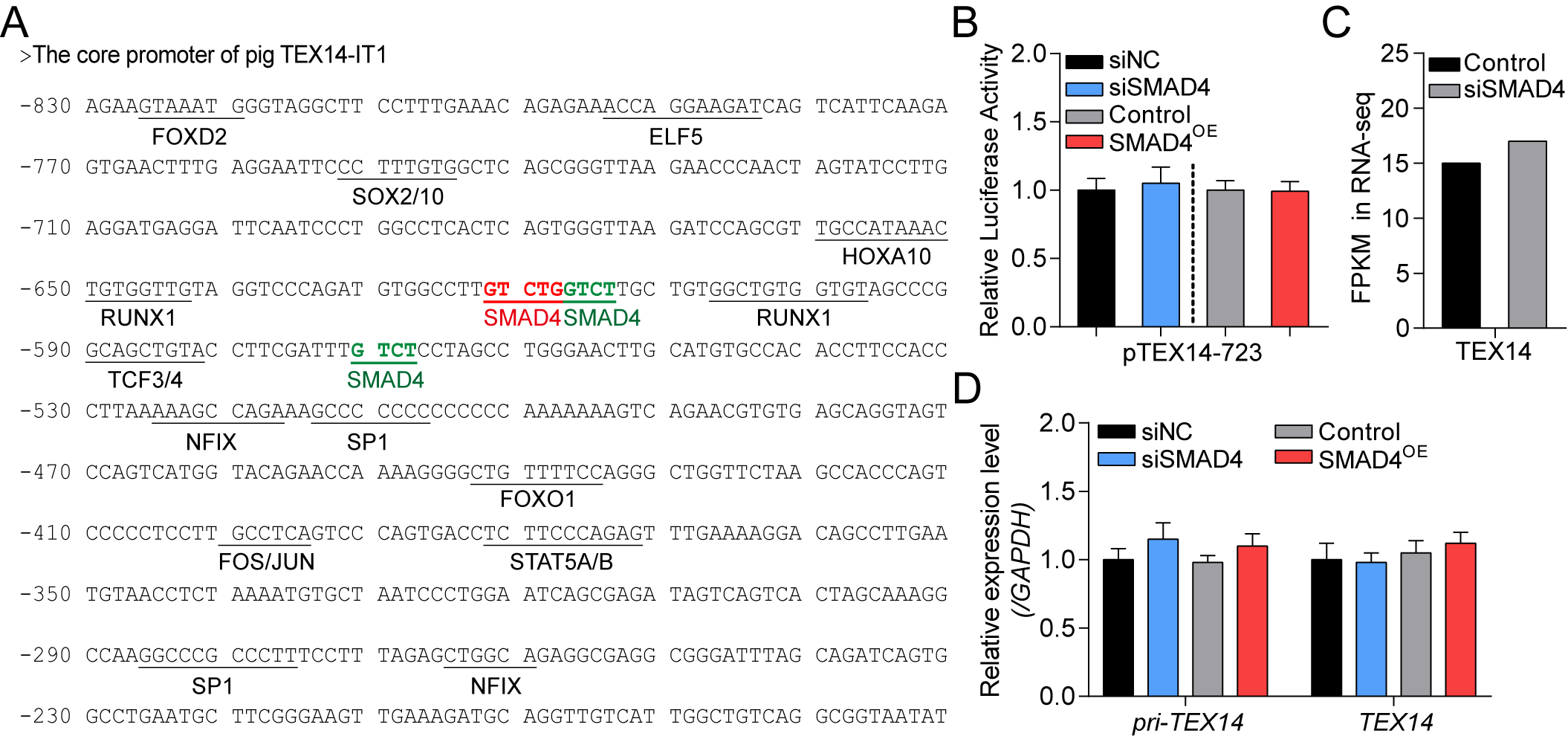


**FIGURE S5** SMAD4 has no effect on the transcription of *TEX14*, the host gene of *TEX14-IT1*. (A) Identification and characterization of the core promoter of pig *TEX14-IT1*. The binding motifs of crucial TFs within the core promoter of *TEX14-IT1* were indicated underlined. The SBE1 with “GTCTG” motif was shown in red and SBE2/3 with “GTCT” motifs were indicated in green. (B) The effects of SMAD4 on the promoter activity of pig *TEX14* were detected by luciferase activity assays (*n*=3). (C) The levels of *TEX14* transcript (FPKM) in porcine GCs after knockdown of SMAD4 according to the RNA-seq data. (D) The effects of SMAD4 on the expression levels of *pri-TEX14* and *TEX14* in porcine GCs were detected by qRT-PCR assays (*n*=3). Data in B and D were shown as mean ± SEM with three independent replications.

**TABLE S1**. The oligonucleotides used in this study.

| **ID** | **Target gene** | **siRNA sequence (5’-3’)** | **Target site** |
| --- | --- | --- | --- |
| NC-siRNA | - | S:UUC UCC GAA CGU GUC ACG UTT | - |
|  |  | AS:ACG UGA CAC GUU CGG AGA ATT |  |
| MSTRG.29961.1  -siRNA-1 | *MSTRG.29961.1* | S:UGU AGU AGG UCC AAG AAG CTT | 79-97 |
|  |  | AS:GCU UCU UGG ACC UAC UAC ATT |  |
| MSTRG.29961.1  -siRNA-2 |  | S:CUG CAC AGU GUG GAG AUU GTT | 974-992 |
|  |  | AS:CAA UCU CCA CAC UGU GCA GTT |  |
| MSTRG.29961.1  -siRNA-3 |  | S:CUG CAU GGU ACU UGG AUG UTT | 1222-1240 |
|  |  | AS:ACA UCC AAG UAC CAU GCA GTT |  |
| MSTRG.26643.2  -siRNA-1 | *MSTRG.26643.2*  (*TEX14-IT1*) | S:UGU GUG GCU AAG ACU AUA CTT | 42-60 |
|  |  | AS:GUA UAG UCU UAG CCA CAC TTT |  |
| MSTRG.26643.2  -siRNA-2 |  | S:CAC UUG CCC UUG AUG AUC GTT | 195-213 |
|  |  | AS:CGA UCA UCA AGG GCA AGU GTT |  |
| MSTRG.26643.2  -siRNA-3 |  | S:CUG AGC GUG AAG CCG GCU CTT | 499-517 |
|  |  | AS:GAG CCG GCU UCA CGC UCA GTT |  |
| MSTRG.102217.11  -siRNA-1 | *MSTRG.102217.11* | S:AGU CUC AAA CUC UCA AUG GTT | 510-528 |
|  |  | AS:CCA UUG AGA GUU UGA GAC UTT |  |
| MSTRG.102217.11  -siRNA-2 |  | S:CAA AGU CCA CUG AUG CAA ATT | 848-863 |
|  |  | AS:UUU GCA UCA GUG GAC UUU GTT |  |
| MSTRG.102217.11  -siRNA-3 |  | S:UUA ACC UCA GUC UAC AGC GTT | 2170-2188 |
|  |  | AS:CGC UGU AGA CUG AGG UUA ATT |  |
| MSTRG.10023.1  -siRNA-1 | *MSTRG.10023.1* | S:GAU CGC UUG AGC CCA GGA GTT | 60-78 |
|  |  | AS:CUC CUG GGC UCA AGC GAU CTT |  |
| MSTRG.10023.1  -siRNA-2 |  | S:CAU UGA UCG CCA GGG UUG ATT | 210-228 |
|  |  | AS:UCA ACC CUG GCG AUC AAU GTT |  |
| MSTRG.10023.1  -siRNA-3 |  | S:CCC CGA UAG AGG AGG ACC GTT | 329-347 |
|  |  | AS:CGG UCC UCC UCU AUC GGG GTT |  |
| MSTRG.18362.7  -siRNA-1 | *MSTRG.18362.7* | S:CAG AGA GUC GCA GAA UCU GTT | 280-298 |
|  |  | AS:CAG AUU CUG CGA CUC UCU GTT |  |
| MSTRG.18362.7  -siRNA-2 |  | S:GCU GAA GUC AGA UCG CGU GTT | 580-598 |
|  |  | AS:CAC GCG AUC UGA CUU CAG CTT |  |
| MSTRG.18362.7  -siRNA-3 |  | S:CAG GUG AGU GUC GAA ACU GTT | 1060-1078 |
|  |  | AS:CAG UUU CGA CAG UCA CCU GTT |  |

**TABLE S2**. The primers used for qRT-PCR assays

| **Gene** | **Primers (5’-3’)** | **Tm (℃)** | **Product length (bp)** |
| --- | --- | --- | --- |
| *MSTRG.10023.1* | F:CCGCACTAAGTTCGGCATC | 58.6 | 230 |
|  | R:CTCCTCTATCGGGGATGGTC |  |  |
| *MSTRG.115672.2* | F:ACAATGATAGGAAGAGCCGACA | 58.7 | 208 |
|  | R:GTGGAGCAGAAGGGCAAAA |  |  |
| *MSTRG.26643.2*  *(TEX14-IT1)* | F:AGAGAACACCGTCTGACTGGAG | 58.1 | 259 |
|  | R:GCTCAGGAGAAAACGCTACG |  |  |
| *MSTRG.122822.4* | F:GGGGCTTCGCTGACTTTC | 60.0 | 303 |
|  | R:GCGGCTCCAAGACTACAGA |  |  |
| *MSTRG.69268.1* | F:CCTAACGGTTGGAGGTGAAG | 59.1 | 230 |
|  | R:CCACGATACTGCCGACCT |  |  |
| *MSTRG.140557.6* | F:CGAAAGATGGTGAACTATGCC | 56.5 | 273 |
|  | R:CGGGCTTCTTACCCATTTA |  |  |
| *MSTRG.22938.1* | F:AGTCCCTGGTAGAGCGAAAG | 61.0 | 303 |
|  | R:CAAACGGGAACCTGTAGCT |  |  |
| *MSTRG.25872.2* | F:CCTTCCCAGCACCGTTCA | 59.8 | 205 |
|  | R:CCCTCCCTCCCACTTACACTC |  |  |
| *MSTRG.29961.1* | F:GGATGCTTGGTTCTGCTCCTT | 60.4 | 476 |
|  | R:GCCAGACGAAAGCAGGGAG |  |  |
| *MSTRG.102217.11* | F:AAGTCTCAAACTCTCAATGGTTCCT | 59.7 | 229 |
|  | R:GTTTTGCTTTCACCTTTTACCATT |  |  |
| *MSTRG.18362.7* | F:GGGGATTGAAATGGGAGGA | 59.1 | 143 |
|  | R:GTTTGCTTATTCCTGCGTCCT |  |  |
| *MALT1* | F:ACTGAAGCTGCTGGGTGAGA | 58.5 | 108 |
|  | R:TTGATTCCTGGAGGGCTGA |  |  |
| *ICAM1* | F:CCCCTAAACACCATCATACCCA | 61.0 | 114 |
|  | R:CACTTCAGTCTTGTGCCAGTGAGT |  |  |
| *IGF1* | F:ATCGTGGATGAGTGCTGCTTC | 60.0 | 179 |
|  | R:TTCTTGTTTCCTGAACTCCCTCTAC |  |  |
| *IGFBP3* | F:AGGGCACGCCAAGGACAG | 61.5 | 134 |
|  | R:TCAGCGTGTCCTCCATTTCC |  |  |
| *ACVR2B* | F:GCCTGGCTGTTCGGTTTGA | 61.0 | 296 |
|  | R:GGCAGGTCGCATCTTCTTGT |  |  |
| *TIRAP* | F:CTCCAGAGCCGCCTACCCT | 61.5 | 94 |
|  | R:GACAGCCTCCTTGACTTGGTGA |  |  |
| *TP53I3* | F:CTCTTGGGAAATGTTCAGGC | 57.1 | 235 |
|  | R:GGTTGACTCCAGCACCTTTG |  |  |
| *BCL2L1* | F:GAATCAGAAGCGGAAACCC | 60.3 | 169 |
|  | R:AAACTCATCGCCCGCCTC |  |  |
| *ZBED1* | F:CTGCACATGCTCCTCAATACC | 58.0 | 298 |
|  | R:GCATCGGGTAGGGCTTGT |  |  |
| *WNT7B* | F:TCGGACGCTGGAACTGCT | 59.7 | 331 |
|  | R:CTGCCCGCCTCGTTGTT |  |  |
| *MTSS1* | F:TGCCTTCTTGGATGCCTTTC | 60.2 | 318 |
|  | R:ATGTCGCCTCGCCCTTTT |  |  |
| *TGFβ-1* | F:GGCACCCCCCACAGCTTATAT | 60.0 | 291 |
|  | R:GTGGGCACTGAGGCGAAAAC |  |  |
| *UCP2* | F:CCCCTGGACACGGCTAAAGT | 60.5 | 240 |
|  | R:TCTGAGCCCTTGGTGTAGAAAT |  |  |
| *MAF1* | F:GCCCCAGCAGACTGAGCA | 60.0 | 265 |
|  | R:TCATCCACGGCGTTCCAC |  |  |
| *GAPDH* | F:GATGGTGAAGGTCGGAGTG | 60.0 | 246 |
|  | R:CGAAGTTGTCATGGATGACC |  |  |
| *U6* | F:TTATGGGTCCTAGCCTGAC | 60.0 | 169 |
|  | R:CACTATTGCGGGTCTGC |  |  |
| *miR-178* | F:CGGGCGGCTTGTTTTAAG | 60.0 | - |
| *miR-186* | F:CGGGCCAAAGAATTCTCCTT | 60.0 | - |
| *miR-211* | F:CGGGCTTCCCTTTGTCATCC | 60.0 | - |
| *miR-370* | F:CGGGCCAGGTCACGTCTCTG | 60.0 | - |
| *miRNA-R* | R:CAGCCACAAAAGAGCACAAT | 60.0 | - |
| *miR-178*  *stem-loop* | CCTGTTGTCTCCAGCCACAAAAGAGCACAATATTTCAGGAGACAACAGGCAGGCAA | - | - |
| *miR-186*  *stem-loop* | CCTGTTGTCTCCAGCCACAAAAGAGCACAATATTTCAGGAGACAACAGGAGCCCAA | - | - |
| *miR-211*  *stem-loop* | CCTGTTGTCTCCAGCCACAAAAGAGCACAATATTTCAGGAGACAACAGGAGGCGAA | - | - |
| *miR-370*  *stem-loop* | CCTGTTGTCTCCAGCCACAAAAGAGCACAATATTTCAGGAGACAACAGGGTAACTG | - | - |

**TABLE S3**. The DElncRNAs in TGF-β1-treated porcine GCs

| **LncRNAs ID** | **FDR** | **Log_2_(FC)** | **Regulation** | **Chr.^1^** | **Location** |
| --- | --- | --- | --- | --- | --- |
| MSTRG.10023.1 | 5.37E-04 | 11.65 | up | 1 | 179,456,983-182,979,103 |
| MSTRG.139845.3 | 3.75E-03 | 10.28 | up | Unknown | 38,626-51,024 |
| MSTRG.10023.3 | 1.95E-03 | 9.86 | up | 1 | 179,456,983-182,979,098 |
| MSTRG.139845.12 | 3.11E-03 | 9.21 | up | Unknown | 48,139-51,076 |
| MSTRG.115672.2 | 3.93E-03 | 8.89 | up | 7 | 10,867,820-10,868,339 |
| MSTRG.139845.4 | 5.36E-03 | 8.45 | up | 1 | 48,130-51,932 |
| MSTRG.139832.6 | 6.26E-03 | 8.24 | up | 1 | 69-9,630 |
| MSTRG.26643.2 | 7.25E-03 | 8.03 | up | 12 | 34,885,721-34,899,646 |
| MSTRG.122822.4 | 9.27E-03 | 7.69 | up | 7 | 116,785,963-116,787,153 |
| MSTRG.81341.6 | 1.04E-02 | 7.53 | up | 3 | 25,187,249-25,191,913 |
| MSTRG.13594.3 | 1.05E-02 | 7.52 | up | 1 | 252,807,140-252,818,545 |
| MSTRG.116007.7 | 1.21E-02 | 7.31 | up | 7 | 16,945,349-17,361,433 |
| MSTRG.139845.6 | 1.26E-02 | 7.25 | up | 13 | 48,130-51,104 |
| MSTRG.137894.10 | 2.38E-02 | 6.36 | up | 9 | 129,179,587-129,248,887 |
| MSTRG.124763.2 | 2.93E-02 | 6.17 | up | 8 | 31,580,478-31,591,642 |
| MSTRG.32683.2 | 3.07E-02 | 5.99 | up | 13 | 65,751,503-65,758,857 |
| MSTRG.96635.1 | 3.29E-02 | 5.89 | up | 5 | 3,294,853-3,301,660 |
| MSTRG.81671.7 | 5.07E-02 | 5.89 | up | 3 | 28,907,910-28,914,966 |
| MSTRG.15108.4 | 3.32E-02 | 5.88 | up | 1 | 273,498,336-273,502,136 |
| MSTRG.13303.3 | 3.85E-02 | 5.67 | up | 1 | 248,406,332-248448,022 |
| MSTRG.133547.4 | 3.87E-02 | 5.66 | up | 1 | 49,098,506-49,111,700 |
| MSTRG.81790.2 | 3.89E-02 | 5.65 | up | 9 | 31,133,429-31,141,584 |
| MSTRG.139832.14 | 3.96E-02 | 5.63 | up | 3 | 69-9,630 |
| MSTRG.80019.4 | 4.01E-02 | 5.61 | up | 3 | 8,961,323-8,972,071 |
| MSTRG.28762.6 | 4.55E-02 | 5.42 | up | 12 | 59,953,771-59,995,228 |
| MSTRG.116823.2 | 4.91E-02 | 5.31 | up | 7 | 27,254,351-27,259,769 |
| MSTRG.139832.28 | 4.93E-02 | 5.31 | up | 13 | 7,000,792-7,030,898 |
| MSTRG.20225.11 | 4.93E-02 | 5.31 | up | 11 | 9,805-11,347 |
| MSTRG.2826.1 | 5.03E-02 | 5.19 | up | 1 | 51,361,863-51,419,977 |
| MSTRG.63597.5 | 5.04E-02 | 5.18 | up | 17 | 41,490,170-41,496,323 |
| MSTRG.115753.7 | 5.04E-02 | 5.16 | up | 7 | 12,722,196-12,728,024 |
| MSTRG.139845.9 | 9.36E-03 | -4.75 | down | 6 | 48,130-51,104 |
| MSTRG.69268.1 | 9.65E-03 | -4.91 | down | 2 | 6,749,808-6,757,177 |
| MSTRG.122184.1 | 5.00E-02 | -5.11 | down | 7 | 100,041,862-100,123,373 |
| MSTRG.15108.1 | 5.00E-02 | -5.12 | down | 1 | 273,498,301-273,501,295 |
| MSTRG.140436.8 | 5.00E-02 | -5.18 | down | Unknown | 1,058,496-1,063,946 |
| MSTRG.13303.2 | 5.00E-02 | -5.18 | down | 1 | 248,406,302-248,448,072 |
| MSTRG.76790.6 | 5.00E-02 | -5.24 | down | 2 | 117,502,576-117,503,821 |
| MSTRG.2826.3 | 5.00E-02 | -5.26 | down | 1 | 51,361,993-51,419,962 |
| MSTRG.111755.15 | 5.00E-02 | -5.33 | down | 6 | 103,116,535-103,133,856 |
| MSTRG.48627.5 | 5.00E-02 | -5.33 | down | 14 | 127,630,450-127,650,093 |
| MSTRG.62676.8 | 4.97E-02 | -5.35 | down | 17 | 32,594,234-32,604,472 |
| MSTRG.54736.13 | 4.68E-02 | -5.44 | down | 15 | 104,809,457-104,835,608 |
| MSTRG.25872.2 | 4.48E-02 | -5.46 | down | 12 | 23,580,382-23,582,156 |
| MSTRG.140554.1 | 1.35E-02 | -5.51 | down | Unknown | 3,281-10,809 |
| MSTRG.45119.7 | 4.07E-02 | -5.65 | down | 14 | 67,593,366-67628,356 |
| MSTRG.102950.8 | 5.01E-02 | -5.87 | down | 5 | 93,252,035-93,258,637 |
| MSTRG.121030.8 | 3.47E-02 | -5.88 | down | 7 | 86,110,062-86,123,762 |
| MSTRG.124763.1 | 3.43E-02 | -5.90 | down | 8 | 31,580,475-31,591,644 |
| MSTRG.13673.7 | 3.41E-02 | -5.91 | down | 1 | 253,553,236-253,555,674 |
| MSTRG.29961.1 | 2.90E-02 | -6.15 | down | 13 | 23,271,235-23,275,971 |
| MSTRG.18362.7 | 2.51E-02 | -6.36 | down | 10 | 48,048,213-48,049,707 |
| MSTRG.122822.5 | 4.70E-02 | -6.45 | down | 7 | 116,785,963-116,787,056 |
| MSTRG.98703.7 | 2.34E-02 | -6.45 | down | 5 | 24,797,524-24,877,647 |
| MSTRG.133547.1 | 2.31E-02 | -6.48 | down | 9 | 49,098,506-49,111,632 |
| MSTRG.102217.11 | 2.18E-02 | -6.67 | down | 5 | 81,848,146-81,854,007 |
| MSTRG.139832.4 | 1.45E-02 | -7.14 | down | 6 | 68-49,036 |
| MSTRG.17668.5 | 1.39E-02 | -7.21 | down | 10 | 41,895,062-42,013,688 |
| MSTRG.139832.12 | 1.29E-02 | -7.32 | down | 13 | 69-49,030 |
| MSTRG.139832.18 | 1.25E-02 | -7.35 | down | 9 | 4,792-7,775 |
| MSTRG.139832.23 | 1.25E-02 | -7.35 | down | 13 | 8,354-9,632 |
| MSTRG.69511.1 | 1.35E-02 | -7.55 | down | 2 | 8,928,984-8,930,884 |
| MSTRG.140557.5 | 7.69E-03 | -8.05 | down | 6 | 11,726-14,637 |
| MSTRG.139833.2 | 7.10E-03 | -8.16 | down | 6 | 111-2,859 |
| MSTRG.139832.21 | 5.87E-03 | -8.43 | down | 1 | 8,352-9,597 |
| MSTRG.139845.14 | 8.06E-03 | -9.17 | down | Unknown | 48,143-51,932 |
| MSTRG.139832.25 | 2.02E-03 | -9.94 | down | 13 | 9,803-11,407 |
| MSTRG.140557.7 | 1.93E-03 | -10.00 | down | 6 | 11,726-14,637 |
| MSTRG.140557.2 | 1.91E-03 | -10.02 | down | 6 | 11,726-14,637 |
| MSTRG.22938.1 | 1.58E-03 | -10.28 | down | 11 | 66,679,056-66,718,575 |
| MSTRG.139845.11 | 6.59E-04 | -11.52 | down | Unknown | 48,139-51,076 |
| MSTRG.140557.6 | 8.72E-05 | -14.37 | down | 6 | 11,726-14,637 |

^1^Chr. indicates chromosome in pig genome.

**TABLE S4**. The targets of DElncRNA in porcine GCs treated with TGF-β1

| **LncRNAs ID** | **Targets** | |
| --- | --- | --- |
|  | **Trans-acting** | **Cis-acting** |
| MSTRG.10023.1 | *SEMA6C, PTPN14, PDE6C, FAM83G, SCN4A, PSRC1, VPS37C, RAB3GAP2, SLC25A34, NECTIN1, MSX1, MAP3K2* | *RPS29* |
| MSTRG.139845.3 | *TMEM190, TAL1, TMEM141, SOWAHD, TMEM247* | *ENSSSCG00000032599* |
| MSTRG.10023.3 | *MYLK, RAB3GAP2, PSRC1, VPS37C, CDK10, CCR9, LAMA3, MSX1, PTPN14, FBXL16, SCN4A, FAM53B, SLC25A34, CHD5, PRR30, TMEM72, MAP3K2, ZNF81, GPRIN1, SLC39A1, TSC22D3*  *SEMA6C, RASGEF1C, ZNF219, PDE6C* | *RPS29* |
| MSTRG.139845.12 | *TAL1, PWWP2B* | *ENSSSCG00000032599* |
| MSTRG.139845.4 | *NRG2, C2orf72, GPR37L1, PPP1R3F, IER5L* | *ENSSSCG00000032599* |
| MSTRG.139832.6 | *TAL1, TMEM190, ZBTB21, PWWP2B, TMEM141, TMEM247* | - |
| MSTRG.26643.2 | *TIRAP, TP53I3* | *ENSSSCG00000039190* |
| MSTRG.122822.4 | *SOX10, BMP6, FAM120A, LAT, SIX5* | *GLRX5* |
| MSTRG.81341.6 | *-* | *ENSSSCG00000022466*  *ENSSSCG00000034074*  *ACSM3, LYRM1* |
| MSTRG.13594.3 | *-* | *-* |
| MSTRG.116007.7 | *-* | *SOX4* |
| MSTRG.139845.6 | *TMEM190, ENSSSCG00000037666, ENSSSCG00000034846, TMEM247, DBP,* | *ENSSSCG00000032599* |
| MSTRG.137894.10 | *-* | *SMYD2* |
| MSTRG.124763.2 | *ZNF576, ID1, APOBEC3B, ENSSSCG00000020870*  *ENSSSCG00000001398, ENSSSCG00000034866, PSMD2*  *BMX, KIF3C, PPIB, MLST8, SLC35B1, DEFB128, MTMR1*  *CADM4, SLC34A2, ENSSSCG00000013427, MYOC, SLC29A1*  *ENSSSCG00000038107, SCNM1, NOTCH3, CD52, BDH1, NUP133*  *CSTF2, PLS3, C19orf60, YIPF5, FUCA1* | *-* |
| MSTRG.32683.2 | *-* | *THUMPD3, SETD5*  *ENSSSCG00000033436* |
| MSTRG.96635.1 | *-* | *CDPF1, TRMU, GTSE1,*  *PPARA, PKDREJ, TTC38* |
| MSTRG.81671.7 | *-* | *ENSSSCG00000040412* |
| MSTRG.15108.4 | *-* | *WDR5, BRD3* |
| MSTRG.13303.3 | *-* | *ENSSSCG00000005436* |
| MSTRG.133547.4 | *-* | *-* |
| MSTRG.81790.2 | *TNFRSF17* | *-* |
| MSTRG.139832.14 | *TAL1, ENSSSCG00000034846, TMEM190, TMEM247, TMEM141* | *ENSSSCG00000032395* |
| MSTRG.80019.4 | *-* | *ENSSSCG00000038901,*  *AP1S1, SERPINE1, IFT22*  *ENSSSCG00000030709,*  *PLOD3, VGF, ZNHIT1,*  *ENSSSCG00000038938* |
| MSTRG.28762.6 | *-* | *SLC47A2, SLC47A1, ALDH3A1* |
| MSTRG.116823.2 | *-* | *GCLC* |
| MSTRG.20225.11 | *ENSSSCG00000004985, NOL7, DEFB128* | *-* |
| MSTRG.139832.28 | *ZBTB2, KRBA1, TMPRSS6* | *ENSSSCG00000032395,*  *ENSSSCG00000032599* |
| MSTRG.2826.1 | *SRI* | *OGFRL1* |
| MSTRG.63597.5 | *-* | *LBP,*  *ENSSSCG00000037783* |
| MSTRG.115753.7 | *SPO11, PLA2G1B, FXN* | *-* |
| MSTRG.139845.9 | *TAL1, TMEM190, ZBTB21, TMEM141, TMEM247* | *ENSSSCG00000032599* |
| MSTRG.69268.1 | *-* | *SSSCA1, LTBP3, KCNK7,*  *ENSSSCG00000012984,*  *FRMD8, FAM89B, EHBP1L1* |
| MSTRG.122184.1 | *POLD2, ENSSSCG00000035655, ENSSSCG00000031692, RIPK2,*  *FBLN1, SBF1, RNF157, NRCAM, TNKS1BP1, HTR6, BRAT1,*  *BSDC1, DGAT1, EYA2, ENHO, DUSP8, JPH4, ELP5, NUAK2*  *PIAS4, PLXND1, ENSSSCG00000040650, SOX8, TRABD* | *LRRC74A, ZDHHC22*  *ENSSSCG00000029760* |
| MSTRG.15108.1 | *PTGES2* | *BRD3, WDR5* |
| MSTRG.140436.8 | *-* | *MAMDC4, TMEM141,*  *EGFL7, LCN15, CCDC183, PHPT1,*  *ENSSSCG00000005835,*  *ENSSSCG00000031768,*  *RABL6, LCN10, LCN8,*  *ENSSSCG00000005834,*  *AGPAT2, EDF1* |
| MSTRG.13303.2 | *TP53I3, TGIF1, INHBA* | *ENSSSCG00000005436* |
| MSTRG.76790.6 | *-* | *ENSSSCG00000034032* |
| MSTRG.2826.3 | *SRI* | *OGFRL1* |
| MSTRG.111755.15 | *CHAC1, TRIM3, PIK3R6* | *-* |
| MSTRG.48627.5 | *INHBA, TGIF1* | *EMX2* |
| MSTRG.62676.8 | *-* | *LZTS3, AVP, OXT*  *MRPS26, UBOX5* |
| MSTRG.54736.13 | *ENSSSCG00000036282, DBT, ENSSSCG00000034735* | *ENSSSCG00000016100,*  *CASP10* |
| MSTRG.25872.2 | *-* | *ARHGAP23, SRCIN1* |
| MSTRG.140554.1 | *-* | *ENSSSCG00000034846* |
| MSTRG.45119.7 | *-* | *-* |
| MSTRG.102950.8 | *-* | *DUSP6* |
| MSTRG.121030.8 | *-* | *-* |
| MSTRG.124763.1 | *ENSSSCG00000034866, FUS, BMX, PSMD2, ID1, ZNF576, APOBEC3B, ENSSSCG00000001398, ENSSSCG00000020870,*  *MTMR1, DEFB128, CADM4, SLC34A2, PPIB, KIF3C, SLC35B1,*  *MLST8, SCNM1, NOTCH3, ENSSSCG00000013427, MYOC*  *ENSSSCG00000038107, SLC29A1, YIPF5,BDH1, CD52, CSTF2,*  *PLS3, CYB5D2, NUP133* | *-* |
| MSTRG.13673.7 | *-* | *ENSSSCG00000005473*  *SLC46A2, SAL1*  *ENSSSCG00000038875* |
| MSTRG.29961.1 | *MALT1, ICAM1, IGFBP3* | *EXOG, XYLB, ACVR2B* |
| MSTRG.18362.7 | *-* | *ENSSSCG00000026354* |
| MSTRG.122822.5 | *ARHGEF40, SOX10, BMP6* | *GLRX5* |
| MSTRG.98703.7 | *TGIF1, INHBA* | *-* |
| MSTRG.133547.1 | *-* | *-* |
| MSTRG.102217.11 | *ENSSSCG00000035208* | *IGF1* |
| MSTRG.139832.4 | *ZBTB21, FZD7, ENSSSCG00000034846, TMEM190,*  *TMEM141, ELFN1, FAM173A* | *ENSSSCG00000032395* |
| MSTRG.17668.5 | *-* | *-* |
| MSTRG.139832.12 | *SOX1, ENSSSCG00000034846, TMEM190* | *ENSSSCG00000032395* |
| MSTRG.139832.18 | *HES5, DMRTB1, PRDX4, ENSSSCG00000035587* | *ENSSSCG00000032395,*  *ENSSSCG00000032599* |
| MSTRG.139832.23 | *BRF2, ATXN2L, KLC3, TFEB, FBXL22, CEP170B*  *HOXA5, CAPN5, HMCN2, KIAA0556* | *ENSSSCG00000032395,*  *ENSSSCG00000032599* |
| MSTRG.69511.1 | *TP53I3, ICAM1* | *STX5, TAF6L, ZBTB3,*  *ENSSSCG00000040074*  *TMEM179B, NXF1, SLC3A2, POLR2G*  *TMEM223, WDR74* |
| MSTRG.140557.5 | *ENSSSCG00000034846, TMEM190, PKD1, TMEM247, TMEM141* | *-* |
| MSTRG.139833.2 | *DIDO1,ENSSSCG00000035683, ENSSSCG00000032395*  *ENSSSCG00000032599, AURKAIP1* | *ENSSSCG00000032395* |
| MSTRG.139832.21 | *FBXL22, TFEB, ATXN2L, BRF2, KLC3, NAA30, KIAA0556,*  *CAPN5, HMCN2, HOXA5, CEP170B* | *ENSSSCG00000032395,*  *ENSSSCG00000032599* |
| MSTRG.139845.14 | *TMEM190, ENSSSCG00000034846, TMEM247, CDK2AP2* | *ENSSSCG00000032599* |
| MSTRG.139832.25 | *PHLPP1* | *ENSSSCG00000032395,*  *ENSSSCG00000032599* |
| MSTRG.140557.7 | *ENSSSCG00000034846, TMEM190, TMEM141, PKD1*  *TMEM247, ZBTB21* | *-* |
| MSTRG.140557.2 | *PKD1,TMEM141, TMEM247, ZBTB45, ENSSSCG00000034846*  *TMEM190* | *-* |
| MSTRG.22938.1 | *-* | *-* |
| MSTRG.139845.11 | *ENSSSCG00000037666, TMEM190, ENSSSCG00000034846*  *TMEM247, TMEM141* | *ENSSSCG00000032599* |
| MSTRG.140557.6 | *TMEM190, ENSSSCG00000034846, PTGER1, ELFN1, UBAC1, ENSSSCG00000033660, ZBTB21, ZBTB45, TMEM141, TMEM247,* | *-* |

**TABLE S5**. GO terms that DElncRNAs were significantly enriched in

| **Category** | **GO term** | **GO name** | ***P*-value** | **Gene No.** |
| --- | --- | --- | --- | --- |
| BP | GO:0045893 | Transcription regulation (positive) | 4.17E-02 | 6 |
| BP | GO:0043066 | Apoptosis regulation (negative) | 4.17E-02 | 6 |
| BP | GO:0030308 | Cell growth regulation (negative) | 4.46E-02 | 6 |
| BP | GO:0045669 | Osteoblast differentiation regulation (positive) | 1.50E-02 | 5 |
| BP | GO:0007613 | Memory | 2.06E-02 | 5 |
| BP | GO:0030509 | BMP signaling pathway | 3.40E-02 | 5 |
| BP | GO:0001701 | In utero embryonic development | 3.61E-02 | 5 |
| BP | GO:0007229 | Integrin signaling pathway | 4.73E-02 | 5 |
| BP | GO:0045454 | Cell redox homeostasis | 1.31E-02 | 4 |
| BP | GO:0009952 | Anterior pattern specification | 1.80E-02 | 4 |
| BP | GO:0030501 | Bone mineralization regulation (positive) | 2.68E-02 | 4 |
| BP | GO:0034446 | Substrate adhesion-dependent cell spreading | 4.01E-02 | 4 |
| BP | GO:0050679 | Epithelial cell proliferation regulation (positive) | 4.39E-02 | 4 |
| BP | GO:0048333 | Cesodermal cell differentiation | 1.87E-02 | 3 |
| BP | GO:0021915 | Neural tube development | 1.88E-02 | 3 |
| BP | GO:0001822 | Kidney development | 4.51E-02 | 3 |
| BP | GO:0014065 | Phosphatidylinositol 3-kinase signaling | 4.77E-02 | 3 |
| BP | GO:0043408 | MAPK cascade regulation | 4.81E-02 | 3 |
| BP | GO:0061028 | Establishment of endothelial barrier | 5.00E-02 | 3 |
| BP | GO:0060236 | Mitotic spindle organization regulation | 3.06E-02 | 2 |
| BP | GO:0014834 | Skeletal muscle regeneration | 4.05E-02 | 2 |
| BP | GO:0002260 | Lymphocyte homeostasis | 5.00E-02 | 2 |
| BP | GO:0090074 | Protein homodimerization regulation (negative) | 5.00E-02 | 2 |
| BP | GO:0033299 | Secretion of lysosomal enzymes | 5.00E-02 | 2 |
| BP | GO:0038031 | Wnt signaling pathway via JNK cascade | 5.00E-02 | 2 |
| MF | GO:0004872 | Receptor activity | 3.08E-02 | 6 |
| MF | GO:0042277 | Peptide binding | 4.33E-02 | 4 |
| MF | GO:0003713 | Transcription coactivator activity | 4.55E-02 | 4 |
| MF | GO:0005185 | Neurohypophyseal hormone activity | 5.00E-02 | 2 |
| CC | GO:0031012 | Extracellular matrix | 1.08E-02 | 8 |
| CC | GO:0043235 | Receptor complex | 1.84E-02 | 6 |
| CC | GO:0015629 | Actin cytoskeleton | 4.25E-02 | 6 |
| CC | GO:0031982 | Vesicle | 1.58E-02 | 4 |
| CC | GO:0008305 | Integrin complex | 4.78E-02 | 3 |

**TABLE S6**. KEGG pathway analysis of DElncRNAs after TGF-β1 treatment

| **KEGG Term** | **Pathways** | ***P*-value** | **Gene No.** | **Percentage %** |
| --- | --- | --- | --- | --- |
| ssc04512 | ECM-receptor interaction | 7.35E-03 | 7 | 1.437371663 |
| ssc04550 | Cell pluripotency regulation | 4.84E-02 | 7 | 1.437371663 |
| ssc05410 | Hypertrophic cardiomyopathy | 2.49E-02 | 6 | 1.232032854 |
| ssc04064 | NF-kappa B signaling pathway | 4.07E-02 | 6 | 1.232032854 |
| ssc04672 | Intestinal immune network | 1.28E-02 | 5 | 1.026694045 |
| ssc05321 | Inflammatory bowel disease | 4.04E-02 | 5 | 1.026694045 |
| ssc04115 | p53 signaling pathway | 4.67E-02 | 5 | 1.026694045 |
| ssc04350 | TGF-beta signaling pathway | 4.11E-02 | 5 | 1.026694045 |
| ssc04390 | Hippo signaling pathway | 4.62E-02 | 4 | 2.197802198 |
| ssc04975 | Fat digestion and absorption | 2.99E-02 | 3 | 1.648351648 |

**TABLE S7**. The negative interactions between DElncRNAs and DEmiRNA in porcine GCs

| **DElncRNAs** | **DEmiRNAs** | **MEF (kcal/mol)** | **Binding site** |
| --- | --- | --- | --- |
| MSTRG.10023.1 | miR-186 | 28.7 | 329-349 |
| MSTRG.10023.3 | miR-186 | 28.7 | 329-349 |
| MSTRG.10023.1 | miR-324 | 34.6 | 160-181 |
| MSTRG.10023.3 | miR-324 | 34.6 | 160-181 |
| MSTRG.10023.1 | miR-211 | 25.8 | 148-169 |
| MSTRG.10023.3 | miR-211 | 25.8 | 148-169 |
| MSTRG.115672.2 | miR-292 | 26.4 | 438-459 |
| MSTRG.115672.2 | novel-miR-370 | 25.8 | 250-272 |
| MSTRG.115672.2 | miR-186 | 23.3 | 494-517 |
| MSTRG.26643.2 | miR-178 | 25.1 | 29-51 |
| MSTRG.140557.6 | miR-371 | 26.8 | 1763-1783 |
| MSTRG.140557.6 | miR-381 | 27.4 | 2560-2582 |
| MSTRG.140557.6 | miR-606 | 20.4 | 2156-2177 |
| MSTRG.22938.1 | miR-22 | 23.5 | 1369-1391 |
| MSTRG.22938.1 | miR-493 | 28.4 | 781-803 |
| MSTRG.69511.1 | miR-321 | 27.2 | 403-425 |
| MSTRG.69511.1 | miR-290 | 21.6 | 177-195 |
| MSTRG.17668.5 | miR-127 | 27.9 | 852-874 |
| MSTRG.18362.7 | miR-370 | 31.5 | 537-559 |

**TABLE S8**. The binding motifs of potential TFs within the core promoter of pig *TEX14-IT1*

| **TF ID** | **TF name** | **Score** | **Relative score** | **Start** | **End** | **Predicted motifs** |
| --- | --- | --- | --- | --- | --- | --- |
| MA0635.1 | BARHL2 | 7.703 | 0.913 | -827 | -818 | AGTAAATGGG |
| MA0847.1 | FOXD2 | 7.183 | 0.906 | -826 | -820 | GTAAATG |
| MA0109.1 | HLTF | 7.122 | 0.932 | -812 | -803 | TTCCTTTGAA |
| MA0136.2 | ELF5 | 11.358 | 0.932 | -794 | -784 | ACCAGGAAGAT |
| MA0109.1 | HLTF | 6.765 | 0.919 | -768 | -759 | GAACTTTGAG |
| MA0081.1 | SPIB | 9.740 | 0.975 | -762 | -756 | TGAGGAA |
| MA0143.3 | Sox2 | 12.638 | 0.976 | -752 | -745 | CCTTTGTG |
| MA0442.1 | SOX10 | 8.910 | 1.000 | -751 | -746 | CTTTGT |
| MA0899.1 | HOXA10 | 9.737 | 0.906 | -660 | -650 | TGCCATAAACT |
| MA0635.1 | BARHL2 | 8.460 | 0.927 | -657 | -648 | CATAAACTGT |
| MA0847.1 | FOXD2 | 7.225 | 0.907 | -656 | -650 | ATAAACT |
| MA0002.2 | RUNX1 | 10.662 | 0.905 | -653 | -643 | AACTGTGGTTG |
| MA0698.1 | ZBTB18 | 13.798 | 0.932 | -639 | -627 | GTCCCAGATGTGG |
| MA0442.1 | SOX10 | 7.094 | 0.919 | -626 | -621 | CCTTGT |
| MA0513.1 | SMAD4 | 11.628 | 0.983 | -622 | -618 | GTCTG |
| MA0513.1 | SMAD4 | 9.8336 | 0.941 | -617 | -614 | GTCT |
| MA0002.2 | RUNX1 | 10.840 | 0.909 | -607 | -597 | GGCTGTGGTGT |
| MA0824.1 | ID4 | 9.230 | 0.902 | -591 | -582 | GGCAGCTGTA |
| MA0522.2 | TCF3 | 8.990 | 0.921 | -591 | -582 | GGCAGCTGTA |
| MA0830.1 | TCF4 | 8.585 | 0.918 | -591 | -582 | GGCAGCTGTA |
| MA0513.1 | SMAD4 | 9.8336 | 0.941 | -571 | -568 | GTCT |
| MA0599.1 | KLF5 | 8.827 | 0.9155 | -545 | -536 | GCCACACCTT |
| MA0090.2 | TEAD1 | 9.361 | 0.916 | -541 | -532 | CACCTTCCAC |
| MA0650.1 | HOXA13 | 10.471 | 0.903 | -532 | -523 | CCCTTAAAAA |
| MA0909.1 | HOXD13 | 9.861 | 0.903 | -532 | -523 | CCCTTAAAAA |
| MA0671.1 | NFIX | 6.852 | 0.913 | -525 | -517 | AAAGCCAGA |
| MA0741.1 | KLF16 | 12.819 | 0.927 | -514 | -504 | GCCCCCCCCCC |
| MA0079.3 | SP1 | 9.760 | 0.903 | -514 | -504 | GCCCCCCCCCC |
| MA0676.1 | Nr2e1 | 11.547 | 0.936 | -497 | -489 | AAAAGTCAG |
| MA0259.1 | HIF1A | 8.047 | 0.905 | -489 | -482 | GAACGTGT |
| MA0004.1 | Arnt | 8.609 | 0.930 | -488 | -483 | AACGTG |
| MA0801.1 | MGA | 11.064 | 0.945 | -487 | -480 | ACGTGTGA |
| MA0805.1 | TBX1 | 11.001 | 0.929 | -487 | -480 | ACGTGTGA |
| MA0806.1 | TBX4 | 10.479 | 0.939 | -487 | -480 | ACGTGTGA |
| MA0807.1 | TBX5 | 9.479 | 0.918 | -487 | -480 | ACGTGTGA |
| MA0745.1 | SNAI2 | 9.182 | 0.931 | -480 | -472 | AGCAGGTAG |
| MA0480.1 | Foxo1 | 11.904 | 0.923 | -445 | -435 | GGCTGTTTTCC |
| MA0606.1 | NFAT5 | 9.553 | 0.904 | -441 | -432 | GTTTTCCAGG |
| MA0152.1 | NFATC2 | 11.360 | 1.000 | -440 | -434 | TTTTCCA |
| MA0719.1 | RHOXF1 | 8.661 | 0.972 | -424 | -417 | CTAAGCCA |
| MA0599.1 | KLF5 | 7.815 | 0.902 | -410 | -401 | CCCCCTCCTT |
| MA0099.2 | FOS:JUN | 8.185 | 0.909 | -401 | -395 | TGCCTCA |
| MA0519.1 | STAT5A/B | 11.589 | 0.935 | -382 | -372 | TCTTCCCAGAG |
| MA0498.2 | MEIS1 | 5.680 | 0.901 | -364 | -358 | AGGACAG |
| MA0648.1 | GSC | 13.398 | 0.999 | -333 | -324 | GCTAATCCCT |
| MA0891.1 | GSC2 | 11.212 | 0.967 | -333 | -324 | GCTAATCCCT |
| MA0714.1 | PITX3 | 12.246 | 0.989 | -333 | -325 | GCTAATCCC |
| MA0711.1 | OTX1 | 11.045 | 0.976 | -332 | -325 | CTAATCCC |
| MA0712.1 | OTX2 | 11.078 | 0.975 | -332 | -325 | CTAATCCC |
| MA0671.1 | NFIX | 7.441 | 0.927 | -294 | -286 | AAGGCCAAG |
| MA0079.3 | SP1 | 10.867 | 0.917 | -286 | -276 | GGCCCGCCCTT |
| MA0161.1 | NFIC | 8.396 | 0.956 | -265 | -260 | CTGGCA |
| MA0745.1 | SNAI2 | 8.791 | 0.923 | -203 | -195 | TGCAGGTTG |
| MA0161.1 | NFIC | 8.520 | 0.960 | -191 | -186 | TTGGCT |
